# Mesodermal gene expression during the embryonic and larval development of the articulate brachiopod *Terebratalia transversa*

**DOI:** 10.1186/s13227-015-0004-8

**Published:** 2015-04-11

**Authors:** Yale J Passamaneck, Andreas Hejnol, Mark Q Martindale

**Affiliations:** Kewalo Marine Laboratory, PBRC, University of Hawaii, 41 Ahui Street, Honolulu, HI 96813 USA; The Whitney Laboratory for Marine Bioscience, University of Florida, St. Augustine, FL 32080 USA; Sars International Centre for Marine Molecular Biology, University of Bergen, Thormøhlensgate, 55, 5008 Bergen, Norway

**Keywords:** Brachiopod, *Terebratalia transversa*, Mesoderm, Spiralia, Ectomesoderm

## Abstract

**Background:**

Brachiopods undergo radial cleavage, which is distinct from the stereotyped development of closely related spiralian taxa. The mesoderm has been inferred to derive from the archenteron walls following gastrulation, and the primary mesoderm derivative in the larva is a complex musculature. To investigate the specification and differentiation of the mesoderm in the articulate brachiopod *Terebratalia transversa*, we have identified orthologs of genes involved in mesoderm development in other taxa and investigated their spatial and temporal expression during the embryonic and larval development of *T. transversa*.

**Results:**

Orthologs of 17 developmental regulatory genes with roles in the development of the mesoderm in other bilaterian animals were found to be expressed in the developing mesoderm of *T. transversa*. Five genes, *Tt.twist*, *Tt.GATA456*, *Tt.dachshund*, *Tt.mPrx*, and *Tt.NK1*, were found to have expression throughout the archenteron wall at the radial gastrula stage, shortly after the initiation of gastrulation. Three additional genes, *Tt.Pax1/9*, *Tt.MyoD*, and *Tt.Six1/2*, showed expression at this stage in only a portion of the archenteron wall. *Tt.eya*, *Tt.FoxC*, *Tt.FoxF*, *Tt.Mox*, *Tt.paraxis*, *Tt.Limpet*, and *Tt.Mef2* all showed initial mesodermal expression during later gastrula or early larval stages. At the late larval stage, *Tt.dachshund*, *Tt.Limpet*, and *Tt.Mef2* showed expression in nearly all mesoderm cells, while all other genes were localized to specific regions of the mesoderm. *Tt.FoxD* and *Tt.noggin* both showed expression in the ventral mesoderm at the larval stages, with gastrula expression patterns in the archenteron roof and blastopore lip, respectively.

**Conclusions:**

Expression analyses support conserved roles for developmental regulators in the specification and differentiation of the mesoderm during the development of *T. transversa*. Expression of multiple mesodermal factors in the archenteron wall during gastrulation supports previous morphological observations that this region gives rise to larval mesoderm. Localized expression domains during gastrulation and larval development evidence early regionalization of the mesoderm and provide a basis for hypotheses regarding the molecular regulation underlying the complex system of musculature observed in the larva.

**Electronic supplementary material:**

The online version of this article (doi:10.1186/s13227-015-0004-8) contains supplementary material, which is available to authorized users.

## Background

The mesoderm is frequently considered the ‘third germ layer’ in metazoans. As its name suggests, the mesoderm is a ‘middle’ layer, located between the ectoderm and endoderm. The mesoderm gives rise to tissues including muscle, parenchyma, cartilage, hemolymph, and somatic gonads, as well as forms the lining of coelomic cavities [1]. The mesoderm is considered to be a unique feature of bilaterian animals, and it is hypothesized that it evolved from the endoderm [2]. The homology of the mesoderm among bilaterians is supported by the deployment of a conserved set of transcription factors in the specification and differentiation of the mesoderm in the classical model systems of the mouse, *Drosophila*, and *Caenorhabditis elegans*, where the molecular basis for mesoderm development has been investigated in the greatest detail [3-6]. Orthologs of the transcription factors Eya [7-9], MyoD [10-12], and Mef2 [13-15] have all been shown to have important roles in mesoderm development and myogenesis in each of these taxa. However, the specific gene regulatory network architecture underlying mesoderm development differs in each of these species, reflecting the significant differences between them in modes of gastrulation, which forms the endomesoderm, and the character and organization of mesodermal derivatives [6,16].

Although mesoderm formation has been studied in detail in these and other model systems, comparatively little is known about mesoderm specification and differentiation in members of the large protostome clade Spiralia [17,18]. This clade is defined as including all descendents from the last common ancestor of animals with quartet spiral cleavage (that is, mollusks, annelids, nemerteans, and platyhelminths) and is likely more inclusive than, or a senior synonym of, the clade termed Lophotrochozoa [19,20]. In spiral cleavage, stereotyped cell divisions result in an invariant developmental program, with tissues and organs in the larva traceable to individual, homologous, blastomeres in the early embryo [21,22]. Almost all taxa that display spiral cleavage form visceral mesoderm from a homologous blastomere in the early cleavage stages termed micromere 4d or the mesentoblast [23]. In addition to this mesendodermal component of the mesoderm, many spiral cleaving embryos also develop mesoderm from ectodermal sources [24,25].

While the internal evolutionary relationships of the Spiralia remain a subject of investigation, nearly all recent phylogenies resolve brachiopods (a.k.a. lamp shells) as having evolved from within the clade for which spiral cleavage was plesiomorphic [20,26-29]. This suggests that brachiopods most likely evolved from an ancestor with spiral cleavage; however, extant brachiopods show no trace of this stereotyped cleavage program [30-33].

**Figure 1:**
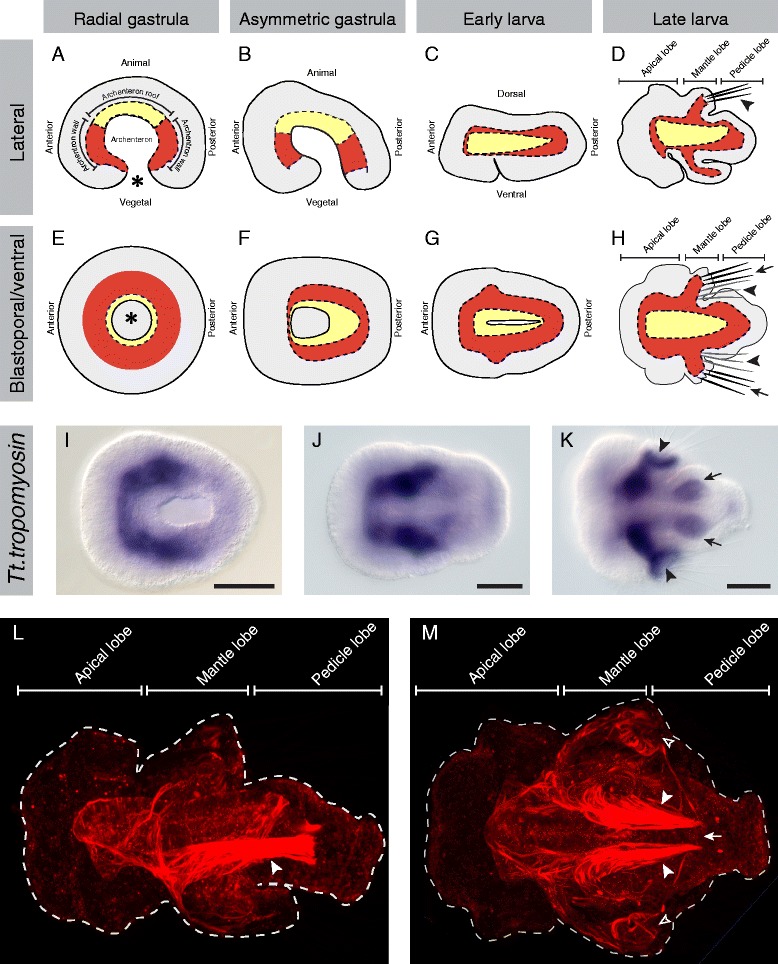
Diagrams of *T*. *transversa* development and distribution of musculature in the competent larva. All images are oriented with anterior to the left. Panels **(A-D)** and **(I)** are lateral views. Panels **(E, F)** and **(J)** are blastoporal/ventral views. Panels **(G, H)** are ventral views. **(A-H)** Diagrammatic views of *T. transversa* gastrula and larval stages. The mesoderm is shaded red, the endoderm is shaded yellow, and the ectoderm is shaded gray. **(A, E)** The location of the blastopore in the radial gastrula stage is denoted by an asterisk. **(D)** Lateral view of the late larval stage. One set of dorsal chaetae (black arrowhead) is shown emerging from the mesodermal chaetal sac in the mantle lobe. **(H)** Ventral view of the late larval stage. Lateral chaetae (black arrows) are shown emerging from the mesodermal chaetal sacs in the mantle lobe. Dorsal chaetae are behind the plane of the cross section (black arrowheads). **(I-K)** Expression of *Tt.tropomyosin* during *T. transversa* development. **(I, J)** *Tt.tropomyosin* is expressed in the anterior and lateral portions of the archenteron wall during late gastrula and early larval stages. **(K)** *Tt.tropomyosin* is expressed in the mesoderm of the mantle lobe, including the chaetal sacs (black arrowheads) and the lateral regions of the pedicle lobe (black arrows) during the late larval stage. Scale bars are 50 μm in length. **(L, M)** Phalloidin staining of filamentous actin in the musculature of the competent larval stage. Images are projections of confocal *z*-series through half of the larva. **(L)** Lateral view showing complex musculature in the apical, mantle, and pedicle lobes. The prominent pedicle muscles can be observed the ventral region of the larva (white arrowhead). **(M)** Ventral view of larval musculature. Bundles of muscles are present in the lateral chaetal sacs of the mantle lobe (open arrowheads). Relatively little staining is detected medially in the pedicle lobe (white arrow) between the paired pedicle muscles (white arrowheads).

Brachiopods develop through a form of radial cleavage, and endomesoderm is formed through invagination of cells at the vegetal pole during gastrulation [34]. Morphological analyses have shown that during early gastrulation, the embryo remains radially symmetrical (Figure 1A,E) and invaginating tissue forming the archenteron extends towards the animal pole [34]. The archenteron consists of two domains, the presumptive endoderm (located on the ‘roof’) and mesoderm located in a ring at the boundary of the ectoderm and endoderm (Figure 1A,E) [33-35]. As gastrulation proceeds, the gastrula becomes asymmetric as the animal and vegetal poles shift positions relative to one another, establishing the anterior-posterior and dorsal-ventral axes (Figure 1B,F). After the blastopore elongates along the ventral side of the embryo, the dorsal surface of the archenteron expands asymmetrically at the boundary of the roof and walls, extending a curtain of cells down towards the ventral side of the embryo [34]. This process generates the mesoderm as a distinct tissue layer, surrounding the endoderm (Figure 1C,G). As the blastopore closes from posterior to anterior, the endoderm seals dorsally to form a sac open to the environment through the remnant of the blastopore anteriorly on the ventral surface of the embryo and closed in a blind ending posteriorly. In the late larval stage, the mesoderm is present in all three main regions of the larva, termed the apical, mantle, and pedicle lobes (Figure 1D,H). Mesodermal differentiation is first indicated by expression of the actin-binding gene *Tt.tropomyosin*, which is expressed in the anterior and lateral regions of the archenteron wall in the asymmetric gastrula and early larval stages (Figure 1I,J). In the late larval stage, *Tt.tropomyosin* is expressed in the mesoderm of the mantle lobe, including the chaetal sacs (black arrowheads in Figure 1K), and in the lateral mesoderm of the pedicle lobe (black arrows in Figure 1K). In the final competent larval form, the predominant mesodermal derivative is a complex system of musculature, which includes prominent longitudinal muscles in the pedicle lobe and two pairs of chaetal sacs in the mantle lobe (Figure 1L,M) [36,37].

The available data from *Terebratalia* suggests that all mesodermal derivatives in the larva are of an endomesodermal origin, derived from cells invaginated at the vegetal blastopore during the radial gastrula stage [34,35,38]. A second source of mesoderm, the ectomesoderm, is present in many other taxa in the clade Spiralia [21,22,39], including in phoronids [40], which are closely related to [20,41-48], or derived from [49-52], brachiopods. To date, no evidence has been presented for an ectodermal source of mesoderm in brachiopods, although detailed lineage analysis of blastomere fates has not been conducted for any member of the group.

In this study, we have analyzed mesoderm development in the articulate brachiopod *Terebratalia transversa* by cloning and examining the spatiotemporal patterns of genes orthologous to ones that have been shown to have roles in mesoderm specification and differentiation in a variety of bilaterian taxa (Additional file 1). These included orthologs of the transcription factor genes *FoxC*, *FoxD*, *FoxF*, *GATA4/5/6*, *MEF2*, *Mox*, *mesoPrx*, *MyoD*, *NK1*, *paraxis*, *Pax1/9*, *Six1/2*, and *twist*, the nuclear protein genes *dachshund*, *eyes absent*, and *Limpet*, and the BMP inhibitor *noggin*. Expression data on these genes provides insight into the molecular basis of mesoderm formation and differentiation in brachiopods and enhances our understanding of potential conservation of mesoderm patterning mechanisms across bilaterian taxa.

## Methods

### Gene cloning and orthology assignment

Genes of interest were chosen based on literature searches for developmental regulators expressed in the mesoderm of diverse bilaterian taxa. Putative homologs of these genes were identified from a *T. transversa* transcriptome using TBLASTN search, followed by reciprocal BLASTX searches against NCBI GenBank. Oligonucleotide primers were designed from recovered contigs for RT-PCR or RACE amplification of genes of interest. PCR amplification was performed on a cDNA library synthesized from mixed-stage embryonic RNA with the Advantage RT-for-PCR Kit (Clontech Laboratories, Inc., Mountain View, CA, USA). RACE amplification was performed on cDNA libraries synthesized from mixed-stage embryonic RNA with the SMARTer RACE Kit (Clontech Laboratories, Inc., Mountain View, CA, USA). Amplified fragments were cloned into pGEM-T vector (Promega, Madison, WI, USA) and verified by Sanger sequencing. Sequences for cloned genes are available in GenBank (accession numbers in Additional file 2). Gene orthology was determined by phylogenetic reconstruction. FASTA-formatted files were generated with the inferred amino acid sequences for cloned genes and representative homologs from other metazoan taxa. Sequence alignment was performed with MUSCLE [53], and resultant alignments were trimmed and corrected by eye to remove non-conserved regions and correct obvious errors. The best-fit likelihood model for each alignment was determined using ProtTest [54]. Phylogenetic reconstruction was performed with MrBayes 3.2 with 4 independent runs of 4 chains and 10,000,000 generations each [55].

### Fertilization and fixation

Adult *T. transversa* (Sowerby 1846) were collected by dredging in San Juan Channel, between San Juan Island and Shaw Island, WA, USA, between October and January in 2008, 2010, and 2012, and were maintained in flow-through seawater aquaria at Friday Harbor Laboratories. *In vitro* fertilization was performed by manual dissection and maceration of gonads. Prior to fertilization, oocytes were maintained in clean seawater until germinal vesicle breakdown and shedding of follicle cells were observed (between 4 and 8 h after stripping of gonads). Sperm were activated with seawater buffered to pH 9.8 with Tris. Following fertilization, embryos were reared in 1-L glass beakers with daily water changes. Embryos were fixed with 4% paraformaldehyde in filtered seawater for 1 h, washed four times in phosphate-buffered saline with 0.1% Tween-20, rinsed with distilled water, and subsequently dehydrated and stored in 100% methanol until *in situ* hybridization.

### *In situ* hybridization

*In situ* hybridization of transcripts for cloned genes was performed using protocols established for chromogenic detection in the cnidarian *Nematostella vectensis* [56]. A detailed protocol is presented in Additional file 3. Hybridization was performed at 62°C for 48 h with DIG-UTP-labeled probes at a concentration of 1 ng/μL. Detection of hybridized probes was performed by staining with NBT and BCIP, after labeling with alkaline phosphatase-conjugated anti-DIG antibody. At least 20 embryos were processed per stage for each gene, and development of staining was checked by a stereomicroscope prior to completion of the *in situ* protocol and mounting for imaging. In all cases, staining was highly consistent within stages. Embryos were cleared and mounted in 80% glycerol, and imaging was performed on a Zeiss AxioSkop microscope equipped with Plan-Apochromat 20×/08 N.A. objective and differential interference contrast optics (Carl Zeiss, Jena, Germany). Images were acquired with a Zeiss AxioCam HRc digital camera and Zeiss AxioVision v4.8 software (Carl Zeiss, Jena, Germany).

## Results

### Phylogenetic analysis and orthology assignment

Full-length or partial cDNA sequences were isolated from *T. transversa* for putative homologs of the transcription factor genes *Forkhead C* (*FoxC*), *Forkhead D* (*FoxD*), *Forkhead F* (*FoxF*), *GATA4/5/6*, *MEF2*, *Mox*, *mesoPrx* (*mPrx*), *MyoD*, *NK1*, *paraxis*, *Pax1/9*, *Six1/2*, and *twist*, the nuclear protein genes *dachshund*, *eyes absent*, and *Limpet*, and the BMP inhibitor gene *noggin*. Orthology assignments were verified by Bayesian analysis of phylogenetics using inferred amino acid sequences of the cloned transcripts and representative sequences from other metazoan taxa (Additional files 4, 5, 6, 7, 8, 9, 10, and 11). *T. transversa* genes are subsequently referred to as *Tt.dachshund*, *Tt.eya*, *Tt.FoxC*, *Tt.FoxD*, *Tt.FoxF*, *Tt.GATA4/5/6*, *Tt.Limpet*, *Tt.MEF2*, *Tt.Mox*, *Tt.mPrx*, *Tt.MyoD*, *Tt.NK1*, *Tt.noggin*, *Tt.paraxis*, *Tt.Pax1/9*, *Tt.Six1/2*, and *Tt.twist*.

### Whole-mount *in situ* expression patterns

**Figure 2:**
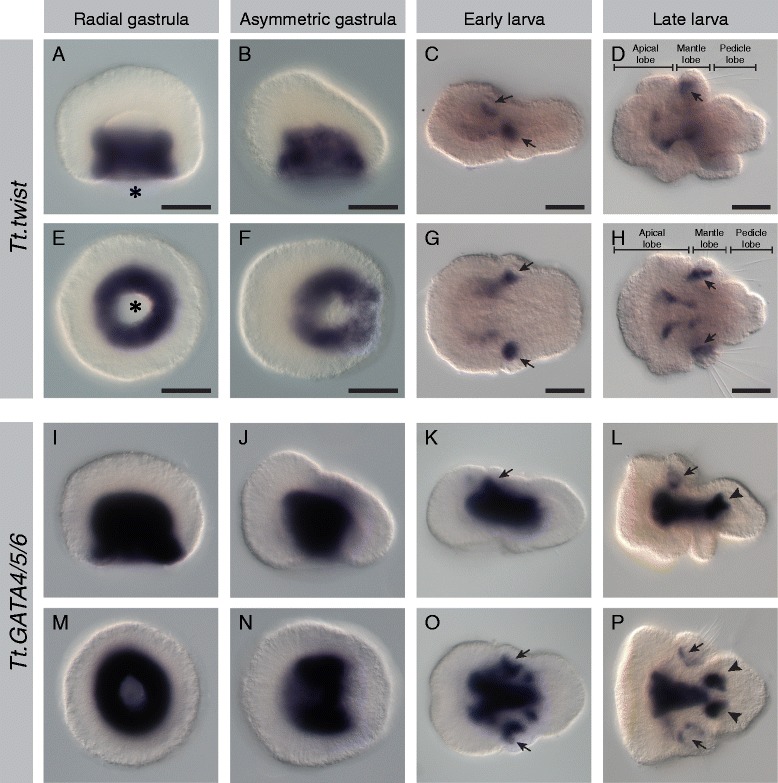
Expression patterns of *Tt*.*twist* and *Tt*.*GATA456*. All images are oriented with anterior to the left. Panels **(A-D)** and **(I-L)** are lateral views. Panels **(E, F)** and **(M, N)** are blastoporal views. Panels **(G, H)** and **(O, P)** are ventral views. For detailed descriptions of expression patterns, see text. **(A-H)** *Tt.twist* expression throughout the archenteron wall at gastrula stages and in the anterior and chaetal sac mesoderm (black arrows) in larval stages. **(A, E)** The location of the blastopore in the radial gastrula stage is denoted by an asterisk. **(I-P)** *Tt.GATA456* expression in the archenteron roof and walls at gastrula stages. Expression in the endoderm, pedicle mesoderm (black arrowheads), and chaetal sac mesoderm (black arrows) in larval stages. Scale bars are 50 μm in length.

**Figure 3:**
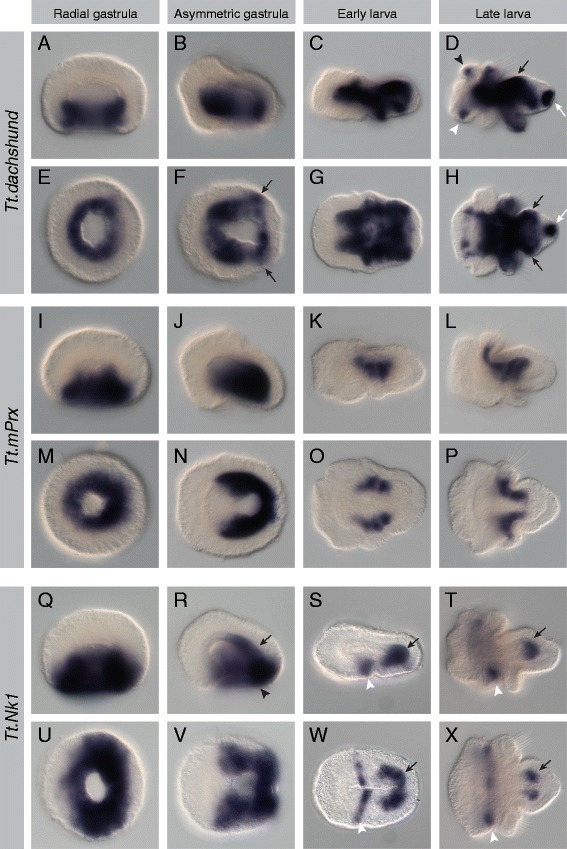
Expression patterns of *Tt*.*dachshund*, *Tt*.*mPrx*, and *Tt*.*NK1*. All images are oriented with anterior to the left. Panels **(A-D)** and **(I-L)** are lateral views. Panels **(E, F)** and **(M, N)** are blastoporal views. Panels **(G, H)** and **(O, P)** are ventral views. For detailed descriptions of expression patterns, see text. **(A-H)** *Tt.dachshund* expression in the archenteron walls at gastrula stages. Broad mesodermal expression at larval stages, along with additional domains in the pedicle lobe ectoderm (black and white arrows) and the dorsal eyespot (black arrowhead) and ventral ganglion (white arrowhead) regions of the apical lobe. **(I-P)** *Tt. mPrx* expression in the archenteron walls at gastrula stages. Expression in the lateral mesoderm at the boundary of the mantle and pedicle lobes in larval stages. **(Q, R, U, V)** *Tt.NK1* expression in the archenteron walls (black arrow) and blastopore lip (black arrowhead) at gastrula stages. **(S, T, W, X)** Expression of *Tt.NK1* in the pedicle mesoderm (black arrow) and posterior apical ectoderm (white arrowhead) at larval stages.

**Figure 4:**
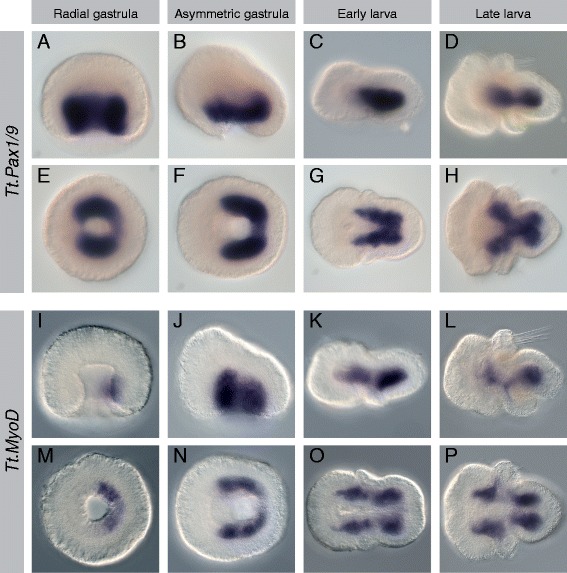
Expression patterns of *Tt*.*Pax1/9* and *Tt*.*MyoD*. All images are oriented with anterior to the left. Panels **(A-D)** and **(I-L)** are lateral views. Panels **(E, F)** and **(M, N)** are blastoporal views. Panels **(G, H)** and **(O, P)** are ventral views. For detailed descriptions of expression patterns, see text. **(A-H)** *Tt.Pax1/9* expression in the lateral and posterior archenteron walls at gastrula stages. Expression in the mesoderm of the mantle and pedicle lobes in larval stages. **(I-P)** *Tt.MyoD* expression in the lateral and posterior archenteron walls at gastrula stages. Expression in the lateral mesoderm of the apical, mantle, and pedicle lobes at larval stages.

**Figure 5:**
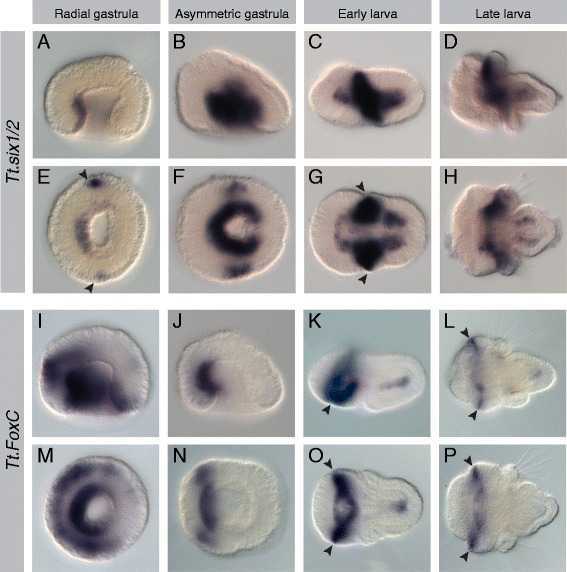
Expression patterns of *Tt*.*Six1/2* and *Tt*.*FoxC*. All images are oriented with anterior to the left. Panels **(A-D)** and **(I-L)** are lateral views. Panels **(E, F)** and **(M, N)** are blastoporal views. Panels **(G, H)** and **(O, P)** are ventral views. For detailed descriptions of expression patterns, see text. **(A-H)** *Tt.Six1/2* is expressed in the archenteron walls and lateral ectoderm (black arrowheads) in the late gastrula stage and in the apical, mantle, and pedicle lobe mesoderm and mantle lobe ectoderm (black arrowheads) in the larval stages. **(I-P)** *Tt.FoxC* expression is first detected in the radial gastrula in the anterior of the archenteron wall and broadly in the adjacent anterior ectoderm. In the asymmetric gastrula, expression persists in the anterior archenteron wall and anterior ectodermal expression has resolved into two lateral bands that extend along the animal-vegetal axis. Ectodermal expression is out of the plane of focus in the lateral view. In larval stages, expression is in the ventral anterior and posterior mesoderm. In the early larval stage, a ventral band of apical ectoderm is positioned near the anterior of the blastopore, and in the late gastrula stage, ectodermal expression is expanded dorsally to form a circumferential ring just anterior of the ciliary band (black arrowheads).

**Figure 6:**
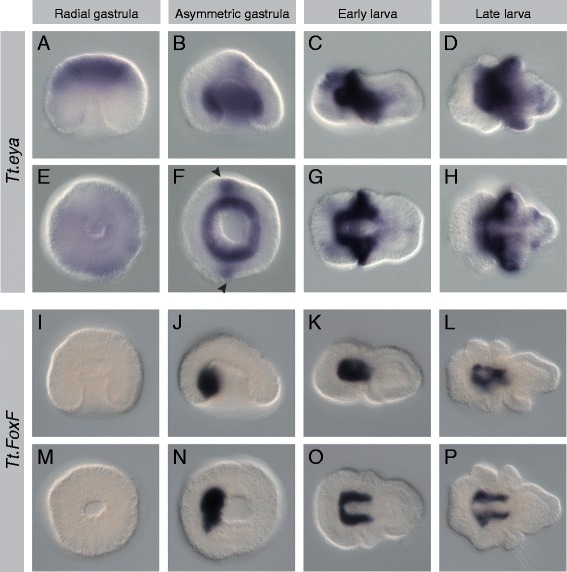
Expression patterns of *Tt*.*eya* and *Tt*.*FoxF*. All images are oriented with anterior to the left. Panels **(A-D)** and **(I-L)** are lateral views. Panels **(E, F)** and **(M, N)** are blastoporal views. Panels **(G, H)** and **(O, P)** are ventral views. For detailed descriptions of expression patterns, see text. **(A-H)** *Tt.eya* is expressed in the animal cap at the radial gastrula stage and in the archenteron walls and lateral ectoderm (black arrowheads) at the asymmetric gastrula stage. *Tt.eya* is expressed in the apical and mantle lobe mesoderm and anterior mantle ectoderm at larval stages. **(I-P)** *Tt.FoxF* expression is first detected in the asymmetric gastrula in the anterior of the archenteron wall. Expression is in the mesoderm laterally and anteriorly flanking the endoderm in larval stages.

**Figure 7:**
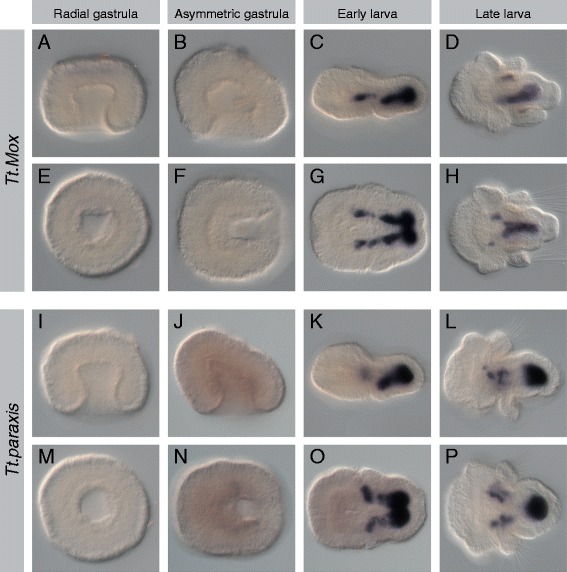
Expression patterns of *Tt*.*Mox* and *Tt*.*paraxis*. All images are oriented with anterior to the left. Panels **(A-D)** and **(I-L)** are lateral views. Panels **(E, F)** and **(M, N)** are blastoporal views. Panels **(G, H)** and **(O, P)** are ventral views. For detailed descriptions of expression patterns, see text. **(A-H)** *Tt.Mox* expression is first detected in the early larva in lateral mesodermal bands flanking the endoderm. Expression in the late larva is in the ventromedial mesoderm. **(I-P)** Expression of *Tt.paraxis* is first detected in the early larva, in the mesoderm of the mantle and pedicle lobes. In the late larva, there are distinct domains of expression underlying the mantle lobe and in the posterior mesoderm of the pedicle lobe.

**Figure 8:**
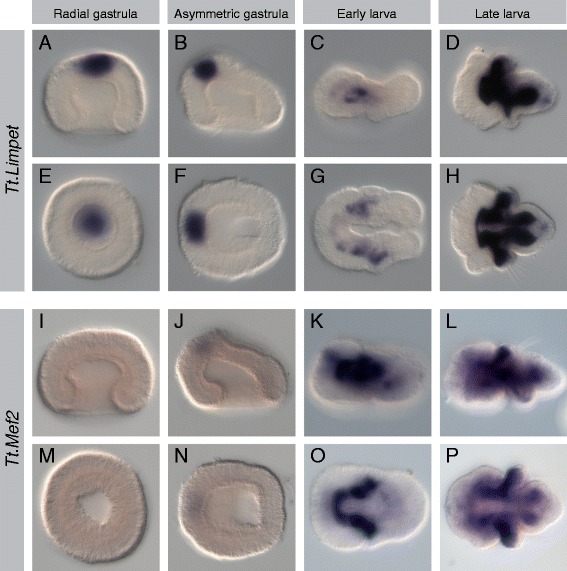
Expression patterns of *Tt*.*Limpet* and *Tt*.*Mef2*. All images are oriented with anterior to the left. Panels **(A-D)** and **(I-L)** are lateral views. Panels **(E, F)** and **(M, N)** are blastoporal views. Panels **(G, H)** and **(O, P)** are ventral views. For detailed descriptions of expression patterns, see text. **(A-H)** *Tt.Limpet* is expressed in the apical ectoderm at the gastrula stages. Mesodermal expression is first observed in the early larva in irregular bands in the developing apical and mantle lobes. In the late larva, strong expression is observed in all but the most posterior region of the mesoderm. **(I-P)** Weak expression of *Tt.Mef2* is observed in the apical ectoderm at the late gastrula stage. In the early larva, a strong continuous band of mesodermal expression flanks the anterior portion of the endoderm and extends laterally into the developing mantle lobe. In the late larva, strong expression is observed flanking the endoderm in the apical lobe and extending into the mantle lobe, including the chaetal sacs. Expression to *Tt.Mef2* is also observed in the pedicle lobe mesoderm.

**Figure 9:**
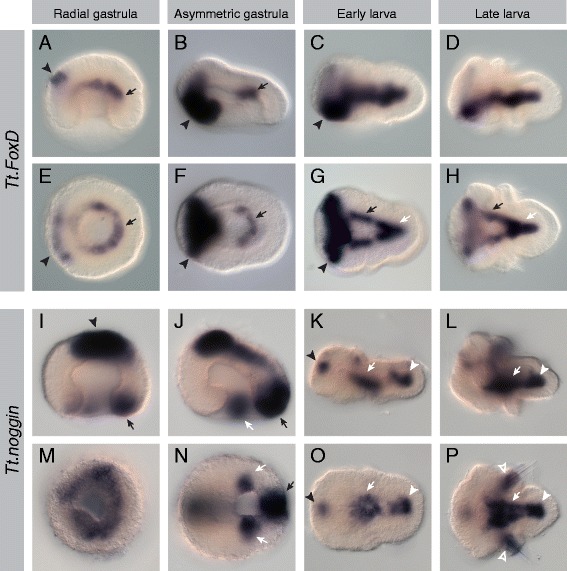
Expression patterns of *Tt*.*FoxD* and *Tt*.*noggin*. All images are oriented with anterior to the left. Panels **(A-D)** and **(I-L)** are lateral views. Panels **(E, F)** and **(M, N)** are blastoporal views. Panels **(G, H)** and **(O, P)** are ventral views. For detailed descriptions of expression patterns, see text. **(A-H)** *Tt.FoxD* is expressed in a narrow band of cells at the border of the archenteron wall and roof in the radial gastrula (black arrows). A second band of expression is present in ectodermal cells at the anterior of the animal half of the embryo (black arrowheads). In the larval stages, *Tt.FoxD* is expressed in two bands of the mesoderm in the mantle lobe (black arrows), which converge ventromedially in the pedicle lobe (white arrows). In the asymmetric gastrula and larval stages, a broad band of ectodermal expression is present on the ventral side just anterior of the blastopore. **(I-P)** *Tt.noggin* is expressed in the blastopore lip (black arrow) and the ectodermal animal cap (black arrowhead) of the radial gastrula. Distinct domains of expression are present in the lateral (white arrows) and posterior (black arrows) regions of the blastopore lip in the asymmetric gastrula. Expression of *Tt.noggin* in the ventral mesoderm of the forming mantle (white arrows) and pedicle lobes (white arrowheads) in the early larva expands to form a single broad domain of expression in the ventromedial mesoderm of the late larva. Additional domains of expression also appear in the ectodermal portions of the chaetal sacs (open white arrowheads) in the late larva.

Mesodermal expression was observed for all 17 genes listed above, and representative photomicrographs of the radial gastrula, asymmetric gastrula, early larval, and late larval stages are presented (Figures 2, 3, 4, 5, 6, 7, 8, and 9). Photomicrographs and descriptions of gene expression patterns are organized in order of the earliest developmental stage when mesodermal expression was detected for each gene. Two other genes with conserved mesodermal expression in other bilaterian taxa, *NK3*/*bagpipe* and *NK4*/*tinman*, were investigated but were not found to have mesodermal expression in the embryonic stages evaluated here (data not shown).

#### Tt.twist

In the radial gastrula, *Tt.twist* is expressed symmetrically throughout the archenteron wall, which is fated to form the mesoderm, but is absent from the archenteron roof, which will form the endoderm (Figure 2A,E). *Tt.twist* continues to be expressed in the archenteron wall at the asymmetric gastrula stage, as the animal pole begins to shift relative to the vegetal pole and the anterior-posterior axis is established (Figure 2B,F). In the early larval stage, *Tt.twist* becomes localized to a horseshoe-shaped domain of the mesoderm surrounding the anterior endoderm in the developing apical lobe (Figure 2C,G). Four additional spots of mesodermal *Tt.twist* expression also appear in the developing mantle lobe of the early larva (black arrows in Figure 2C,G). These paired dorsal and lateral domains correspond to the positions of chaetal sac formation. In the late larva, *Tt.twist* expression remains in the anterior mesoderm and the chaetal sacs (Figure 2D,H).

#### *Tt.GATA4*/*5*/*6*

In the radial gastrula, *Tt.GATA4*/*5*/*6* is expressed in both the mesodermal archenteron wall and the endodermal archenteron roof (Figure 2I,M). In the asymmetric gastrula, *Tt.GATA4*/*5*/*6* expression persists in the archenteron roof and the lateral domains of the archenteron wall, but is absent from the anterior and posterior regions of the archenteron wall (Figure 2J,N). In the early larva, *Tt.GATA4*/*5*/*6* expression in the endoderm becomes localized to the developing midgut, and mesodermal expression is in two paired domains, laterally flanking the anterior and posterior ends of the endoderm (Figure 2K,O). Four additional spots of mesoderm expression form laterally and dorsally in the developing chaetal sacs in the mantle lobe (black arrows in Figure 2K,O). In the late larva, *Tt.GATA4*/*5*/*6* expression persists in the midgut, in two paired mesodermal domains adjacent to the anterior and posterior endoderm (black arrowheads in Figure 2L,P), and in the chaetal sacs (black arrows in Figure 2L,P).

#### Tt.dachshund

In the radial gastrula, *Tt.dachshund* is expressed throughout the mesodermal region of the archenteron wall (Figure 3A,E). In the asymmetric gastrula, *Tt.dachshund* is expressed in the archenteron wall, as well as in the lateral ectoderm adjacent to the posterior end of the archenteron (black arrows in Figure 3F). In the early larva, *Tt.dachshund* is expressed throughout the mesoderm, with the exception of the most anterior and posterior regions of the tissue (Figure 3C,G). A broad contiguous band of expression is also present in the dorsal and lateral ectoderm of the developing pedicle lobe (Figure 3C,G). In the late larva, *Tt.dachshund* shows continued expression in nearly all the mesoderm, including the chaetal sacs and the belt of mesoderm in the extended mantle lobe (Figure 3D,H). Four ectodermal domains of *Tt.dachshund* expression are present in the late larva: in punctate spots in the region of the eyespots on the dorsal side of the apical lobe (black arrowhead in Figure 3D), in punctate spots in the region of the ganglion on the ventral side of the apical lobe (white arrowhead in Figure 3D), in a dorsolateral saddle on the anterior half of the pedicle lobe (black arrows in Figure 3D,H), and in the posterior end of the pedicle lobe (white arrow Figure 3D,H).

#### Tt.mPrx

In the radial gastrula, *Tt.mPrx* is expressed throughout the archenteron wall, with slightly stronger expression in the posterior compared to the anterior (Figure 3I,M). In the asymmetric gastrula, *Tt.mPrx* is expressed in a horseshoe-shaped band of cells covering the lateral and posterior sides of the archenteron wall and is absent from the anterior archenteron wall (Figure 3J,N). In the early larva, *Tt.mPrx* is expressed in two lateral mesodermal bands, flanking the endoderm in the region of the developing apical lobe (Figure 3K,O). In the late larva, *Tt.mPrx* expression laterally flanks the endoderm in the anterior region of the pedicle lobe and extends dorsolaterally into the pedicle lobe, contacting the chaetal sacs (Figure 3L,P).

#### Tt.NK1

In the radial gastrula, *Tt.NK1* is expressed throughout the archenteron walls and blastopore lip and extends laterally into the vegetal ectoderm (Figure 3Q,U). In the asymmetric gastrula, *Tt.NK1* is expressed in the lateral and posterior archenteron walls (black arrow in Figure 3R) and blastopore lip (black arrowhead in Figure 3R) and extends laterally into the vegetal ectoderm adjacent to the blastopore lip (Figure 3R,V). Expression of *Tt.NK1* is absent from the anterior archenteron wall and blastopore lip at this stage. In the early larval stage, *Tt.NK1* is expressed in a crescent of mesoderm around the posterior of the endoderm in the developing pedicle lobe (black arrow in Figure 3S,W) and in a chevron in the ventral ectoderm just anterior of the furrow forming between the apical and mantle lobes (white arrowhead in Figure 3S,W). In the late larva, *Tt.NK1* is expressed in bilateral regions of ectoderm laterally flanking the posterior endoderm in the pedicle lobe (black arrow in Figure 3T,X). A band ectodermal expression is present in the ventral ectoderm at the posterior margin of the apical lobe (white arrowhead in Figure 3T,X).

#### Tt.Pax1/9

In the radial gastrula, *Tt.Pax1/9* is expressed strongly in the lateral sides of the archenteron wall (Figure 4A,E). It is expressed more weakly in the posterior of the archenteron wall and is absent from the anterior of the archenteron wall. In the asymmetric gastrula, *Tt.Pax1/9* is expressed in the ventral portion of the lateral and posterior archenteron wall (Figure 4B,F). In the early larvae, *Tt.Pax1/9* is expressed in a broad V-shaped mesodermal domain, lateral and ventral to the endoderm (Figure 4C,G). In the late larva, *Tt.Pax1/9* expression expands into a Y-shaped domain, with two large regions lateral and ventral to the posterior end of the endoderm, a broad band of expression ventral to the midgut, and two broad domains extending laterally in the mantle lobe (Figure 4D,H).

#### Tt.MyoD

At the radial gastrula stage, *Tt.MyoD* expression is restricted to the posterior wall of the archenteron (Figure 4I,M). By the asymmetric gastrula stage, expression has expanded to a horseshoe shape and includes the lateral archenteron walls (Figure 4J,N). In the early larva, *Tt.MyoD* extends in two lateral bands, extending from the apical lobe to the pedicle lobe and flanking the endoderm (Figure 4K,O). Expression is strongest at the boundary of the apical and mantle lobes and the anterior of the pedicle lobe and is absent from the most posterior medial portion of the mesoderm. The anterior and posterior expression domains persist in the late larvae and are connected by a weaker band of expression that extends ventrally into the mantle lobe (Figure 4L,P).

#### Tt.Six1/2

Expression of *Tt.Six1/2* is first detected at the radial gastrula stage in a narrow band in the anterior mesodermal region of the archenteron wall (Figure 5A,E). At this stage, two additional spots of expression are also observed in the lateral ectoderm (black arrowheads in Figure 5E). By the asymmetric gastrula stage, *TtSix1/2* expression has expanded to all but the most posterior portion of the archenteron wall (Figure 5B,F). In the early larvae, *Tt.Six1/2* is strongly expressed in the mesoderm and ectoderm at the anterior border of the forming mantle lobe (black arrowheads in Figure 5G) and more weakly expressed in the mesoderm of the apical and pedicle lobes (Figure 5C,G). In the late larvae, *Tt.Six1/2* expression remains strong in the mesoderm and ectoderm at the anterior of the mantle lobe and weaker in the apical and pedicle mesoderm (Figure 5D,H).

#### Tt.FoxC

Expression of *Tt.FoxC* is first detected at the radial gastrula stage in the anterior of the archenteron wall and in a broad band in the anterior ectoderm (Figure 5I,M). At the asymmetric gastrula stage, mesodermal expression of *Tt.FoxC* remains localized to the anterior archenteron wall, and ectodermal expression forms two lateral anterior bands (Figure 5J,N). Two bands of expression are also observed in the adjacent anterior lateral ectoderm. In the early larva, there are two mesodermal domains of *Tt.FoxC* expression. In the apical lobe, a dorsal crescent of expression extends laterally, just anterior of the endoderm (Figure 5K,O). In the pedicle lobe, a medial band of expression extends ventrally, below the endoderm. The ventral ectodermal domains of expression are expanded and converge medially at the mouth (black arrowhead in Figure 5K). In the late larva, two dorsolateral domains of mesodermal expression remain at the anterior edge of the endoderm (Figure 5L,P). Ectodermal expression is circumferential at the anterior edge of the ciliary band in the apical lobe.

#### Tt.eya

In the radial gastrula, *Tt.eya* is weakly expressed in the animal cap (Figure 6A,E). Mesodermal expression of *Tt.eya* is first detected at the asymmetric gastrula stage throughout the archenteron wall, as well as in the lateral bands of the ectoderm (black arrowheads in Figure 6F). In the early larva, *Tt.eya* is strongly expressed in a U-shaped domain surrounding the anterior endoderm and in the dorsolateral anterior ectoderm of the developing mantle lobe (Figure 6C,G). Weaker ectodermal bands of expression are also observed dorsolaterally in the apical lobe. Strong expression in the anterior mesoderm and the dorsal anterior mantle ectoderm persists in the late larva (Figure 6D,H). Weaker ectodermal expression is also observed in the ventral half of the mantle lobe and laterally in the pedicle lobe.

#### Tt.FoxF

Expression of *Tt.FoxF* is first observed at the asymmetric gastrula stage in the anterior archenteron wall (Figure 6J,N). In the early larva, a U-shaped domain of mesodermal expression surrounds the anterior endoderm (Figure 6K,O). In the late larva, two lateral bands of mesodermal expression laterally flank the endoderm in the apical and pedicle lobes, with a weak band of expression connecting the anterior of the endoderm (Figure 4L,P).

#### Tt.Mox

Expression of *Tt.Mox* is first observed at the early larval stage (Figure 7C,G). Two domains of strong expression are observed in the posterior mesoderm, with weaker bands of expression laterally flanking the endoderm and extending anteriorly to the apical lobe. In the late larva, a medial band of expression in the posterior mesoderm extends ventrally below the posterior endoderm (Figure 7D,H). Two small domains of expression flank the endoderm at the boundary of the apical and mantle lobes.

#### Tt.paraxis

Expression of *Tt.paraxis* is first observed at the early larval stage (Figure 7K,O). As for *Tt.Mox*, two domains of strong expression are observed in the posterior mesoderm, with weaker expression extending anteriorly, terminating with two lateral mesodermal bands at the boundary of the apical and mantle lobes. In the late larva, a strong domain of expression persists at the posterior mesoderm (Figure 7L,P). Two disjunct and weaker domains of expression are also observed laterally at the boundary of the apical and mantle lobes.

#### Tt.Limpet

In the radial and asymmetric gastrula stages, *Tt.Limpet* expression is exclusively ectodermal, in the central region of the animal cap, where the ciliary apical tuft is located (Figure 8A,B,E,F). In the early larva, expression of *Tt.Limpet* is absent from the ectoderm, and weak expression is detected in the anterior and lateral mesoderm of the developing apical and mantle lobes (Figure 8C,G). In the late larva, expression of *Tt.Limpet* is expressed in nearly all regions of the mesoderm, surrounding the endoderm, extending into the mantle lobe, and forming two large lateral domains in the pedicle lobe (Figure 8D,H). The only region of the late larva mesoderm lacking *Tt.Limpet* expression is in the posterior region of the pedicle lobe, where *Tt.paraxis* is expressed.

#### Tt.Mef2

*Tt.Mef2* is first detected in the asymmetric gastrula, when it is weakly expressed in the apical ectoderm (Figure 8J,N). At the early larval stage, strong expression is detected in the mesoderm of the developing apical and mantle lobes (Figure 8K,O). In the late larva, strong expression persists in the lateral mesoderm of the apical and mantle lobes, and lateral mesoderm expression is also detected in the pedicle lobe (Figure 8L,P).

#### Tt.FoxD

*Tt.FoxD* is expressed at the radial gastrula stage in a narrow band of cells at the border of the archenteron wall and roof in the radial gastrula (black arrows in Figure 9A,E). A second band of expression is present in ectodermal cells at the anterior of the animal half of the embryo at this stage (black arrowheads in Figure 9A,E). In the asymmetric gastrula, endomesodermal expression of *Tt.FoxD* remains in the posterior boundary of the archenteron roof and wall (black arrows in Figure 9B,F). Strong ectodermal expression is observed in a band on the ventral side, just anterior of the blastopore (black arrowheads in Figure 9B,F). In the later bilateral gastrula stage, during which the blastopore becomes elongate and then closes from posterior to anterior, the *Tt.FoxD* expression domain in the archenteron becomes positioned more ventrally and extends anteriorly (black arrows in Additional file 12B,C,F,G). An additional domain of expression also develops ventromedially in the posterior of the archenteron (white arrows in Additional file 12B,C,F,G). In the larval stages, expression is observed in two bands of ventral mesoderm in the mantle lobe (black arrows in Figure 9G,H), which connect to a ventromedial domain of expression in the pedicle lobe (white arrows in Figure 9G,H). A band of ectodermal expression in the ventral portion of the apical lobe persists through larval development (Figure 9C,D,G,H).

#### Tt.noggin

*Tt.noggin* is expressed in the radial gastrula in the blastopore lip (Figure 9M), with prominent expression in the posterior region (black arrow in Figure 9I). Strong ectodermal expression is also observed in the animal cap (black arrowhead in Figure 9I). By the asymmetric gastrula stage, expression in the blastopore lip has resolved into three distinct domains, a region of strong expression in the posterior of the blastopore lip (black arrows in Figure 9J,N) and two smaller domains in the lateral regions of the blastopore lip (white arrows in Figure 9J,N). In the later bilateral gastrula stage, lateral domains of *Tt.noggin* expression in the blastopore lip converge along the midline and shift in from the ventral surface of the embryo as the blastopore elongates and closes (white arrows in Additional file 12 J, K, N, and O). Similarly to *Tt.FoxD*, an additional domain of expression also develops medially in the posterior of the archenteron (white arrowheads in Additional file 12 J, K, N, and O). During the bilateral gastrula stage, the *Tt.noggin* expression domain shifts to the dorsal ectodermal surface of the embryo and decreases in intensity (black arrows in Additional file 12 J and K). In the early larval stage, two domains of *Tt.noggin* expression are observed in the ventral mesoderm of the developing mantle and pedicle lobes (white arrows and white arrowheads, respectively, in Figure 9K,O). A small domain of ectodermal expression is observed in the anterior of the apical lobe (black arrowheads in Figure 9K,O). In the late larva, a single domain of expression in the ventral mesoderm extends from the pedicle lobe to the posterior edge of the apical lobe (white arrowheads and white arrows in Figure 9L,P). Additional expression of *Tt.noggin* is observed in the ectodermal portion of the chaetal sacs (open white arrowheads in Figure 9L,P).

## Discussion

In the present study, we have detected mesodermal expression for 17 developmental regulator genes during the embryonic and larval stages of development in the articulate brachiopod *T. transversa*. Each of these genes shows a unique pattern of expression with regard to both their spatial and temporal deployment, suggesting the dynamic mechanisms underlying the development of the complex larval musculature.

### Expression in the radial gastrula

Five transcription factor genes, *twist*, *GATA456*, *dachshund*, *NK1*, and *mPrx*, showed expression in the whole archenteron wall in the radial gastrula stage. An additional four genes, *Pax1/9*, *MyoD*, *Six1/2*, and *FoxC*, showed localized expression in a portion of the archenteron wall at this stage. Expression in the archenteron wall is consistent with previous morphological observations that this region gives rise to the mesoderm in the larva [34,35]. These genes are therefore all expressed mesodermally during gastrulation and likely play roles in later aspects of mesoderm specification and determination. For *twist* and *GATA456*, comparison with expression and functional data from other bilaterian taxa suggests that these genes are widely utilized in mesoderm specification and differentiation (Additional file 1). Both genes are expressed in the larval mesoderm of the annelids [57-61] and the mesodermal parenchyma of planarian embryos [62,63]. Interestingly, while a *twist* ortholog is expressed in the developing mesoderm of the mollusk *Patella*, it is localized to the ectomesoderm [64], rather than the endomesoderm as it is in *Terebratalia*. Both genes are also involved in the development of mesoderm in ecdysozoans [65-69], although the role of *GATA456* in the arthropod *Drosophila* is limited to the development of the heart [68], a structure which in brachiopods forms only in juveniles after metamorphosis. *Twist* orthologs are also involved in the multiple aspects of mesoderm development in deuterostomes [70-73]. The expression of both *twist* and *GATA456* orthologs in the acoel *Isodiametra* [74] suggests that these genes may have ancestral roles in mesoderm development among bilaterian animals.

The available data is less conclusive for the other three early panmesodermal genes, *dachshund*, *mPrx*, and *NK1* (Additional file 1), although each of these genes is expressed in the developing mesoderm of some taxa. Expression of *Tt.NK1* in the developing pedicle musculature shows similarities to segmental expression in the annelid *Platynereis* [75] and a subset of the somatic musculature in *Drosophila* [76,77]. These results support a role for *NK1* in myogenesis among protostomes, derived from a more ancestral role in ectodermal or neural patterning, which is shared between protostomes and deuterostomes [75,76,78-80]. In the case of *mPrx*, expression patterns have not been described for other protostome taxa. However, broad expression in the developing mesoderm of a hemichordate [81] and mouse [82-84] suggests that greater taxonomic sampling may reveal a previously unrecognized conservation of this gene’s participation in the formation of mesoderm. The case of *Tt.dachshund* is intriguing because of its mesodermal broad expression not only during the gastrula stages but also throughout the larval development. In the annelid *Neanthes*, expression of dachshund is also observed in the mesoderm but only during the initial formation of new segments at the posterior growth zone [85]. While mesodermal expression of dachshund is also reported from deuterostome taxa [86-89], this seems to be derived from a conserved ancestral role in neural development [90,91]. It therefore appears that dachshund has been recruited to play a novel role in mesoderm formation in among brachiopods and annelids and may play an additional role in maintenance of mesodermal identity or mesodermal differentiation in *Terebratalia*.

For those genes that first show regionalized expression in the archenteron wall, there is good evidence that both *MyoD* and *FoxC* have widespread roles in mesoderm development among bilaterians (Additional file 1). *FoxC* is particularly intriguing, as it is expressed in the anterior and posterior mesoderm of annelids [92], mollusks [92], and arthropods [93]. This may be indicative of an evolutionarily conserved role for *FoxC* in patterning mesoderm at the anterior and posterior extremities. *Six1/2* is broadly expressed in the mesoderm in several deuterostomes [81,94,95] and the acoel *Isodiametra* [74], but among protostomes, mesodermal expression has only been reported from *C. elegans*, where it is restricted to the non-muscle coelomocyte lineage [96]. The restriction of *Six1/2* expression to the eyes of an annelid [97], platyhelminth [91], and arthropod [98] suggests that its expression in the mesoderm of *Terebratalia* may be an independent evolutionary acquisition. In the case of *Pax1/9*, mesodermal expression in *Drosophila* [99] and mouse [100,101] is likely acquired independently in the two lineages, given its restriction to the pharyngeal endoderm of more basally divergent deuterostomes [102-104]. However, taxonomic sampling is insufficient to infer a potentially conserved role in protostome mesoderm.

### Expression in the asymmetric gastrula

Mesodermal expression of two genes, *Tt.eya* and *Tt.FoxF*, was first detected in the asymmetric gastrula stage. This later onset of expression suggests that these two genes likely function in a later stage of specification or differentiation than the genes discussed above and may be downstream of them in the mesodermal gene regulatory network (GRN).

Although *Tt.eya* is strongly expressed throughout the archenteron wall in the asymmetric gastrula and in the anterior mesoderm of the larval stages, there is limited evidence for mesodermal expression in other members of the Spiralia. Expression of an *eya* ortholog has been reported from the mesodermally derived photophore (or light organ) of the bobtail squid *Euprymna scolopes* [105]. However, this structure is an evolutionary novelty within cephalopod mollusks, and expression of *eya* appears to be part of a redeployment of the Pax-Six-Eya-Dachshund network from eye specification to facilitate acquisition of light sensitivity in the photophore [105]. In the platyhelminth *Schmidtea*, expression of *eya* is restricted to the eyes during embryonic development and regeneration [91,106]. A mesodermal function for *eya* is more widely reported in ecdysozoans and deuterostomes, with orthologs playing key roles in myogenesis in both *Drosophila* [107] and vertebrates [108] and showing expression in the invaginating endomesoderm in the cephalochordate *Branchiostoma* [94].

The restriction of *Tt.FoxF* expression to the anterior mesoderm of the asymmetric gastrula and larval stages of *Terebratalia* may be comparable to expression in the anterior mesoderm of the mollusk *Patella* [92]. However, in both *Patella* and the annelid *Capitella*, there was also expression in the posterior mesoderm [92], for which no equivalent was observed in *Terebratalia*. More broadly, the expression of *Tt.FoxF* surrounds the larval endoderm, which may share an evolutionary origin with expression in the visceral mesoderm of *Drosophila* [109], the hemichordate *Saccoglossus* [110], and the mouse [111].

### Expression in the larval stages

*Tt.Mox* and *Tt.paraxis* show very similar expression patterns in the mantle and pedicle lobes of the larval stages. *Mox* expression in the ventral mesoderm appears to be a conserved feature in Spiralia, given that orthologs in the annelid *Platynereis* [112] and the mollusk *Haliotis* [113] show comparable expression. Conserved expression of *Mox* is further supported by expression of orthologs in the ventral mesoderm of *Drosophila* [114] and the hemichordate *Saccoglossus* [115]. In chordates, Mox also shows mesodermal expression, although primarily in the paraxial mesoderm [116-118]. Less taxa have been sampled for expression of *paraxis*; however, most available data support a conserved role in mesoderm development. Comparable to the expression of *Tt.paraxis*, in the annelid *Platynereis*, *paraxis* is in the ventrolateral mesoderm [112], and in the cephalochordate *Branchiostoma*, *paraxis* is in developing somites as they form at the posterior of the embryo [119]. In mouse, there are two paralogs, *paraxis*, which is required in somite formation [120], and *scleraxis*, which is required for the initial specification of mesoderm as well as for subsequent chondrogenesis in tissues derived from the somites [121]. The one exception to this trend of mesodermal expression and function for paraxis orthologs is in *Drosophila*, where expression of the ortholog *CG33557* (previously *CG12648*) is restricted to neural cells [122].

Mesodermal expression of *Tt.Limpet* and *Tt.Mef2* is very similar with both first detected anteriorly in the early larva and then expanding throughout nearly all mesodermal tissues by the late larval stage. These patterns of expression suggest that both genes likely have roles in myogenic differentiation, as their localization closely matches that of musculature labeled by phalloidin in slightly older competent larvae. This included the notable absence of expression in the medial and posterior mesoderm where there is no apparent muscle formation. Expression has not been described of either of these genes on taxa in the Spiralia, but the myogenic function of *Mef2* has been well characterized in both *Drosophila* [13,123]. Myogenic roles have also been described for paralogs in mouse [15], and an ancestral function in myogenesis is further supported by expression in the musculature of the acoel *Isodiametra* [74]. Two exceptions to this trend of a myogenic role for *Mef2* are its apparent lack of a developmental function in *C. elegans* [14] and its restriction to non-myogenic mesoderm in the developing sea urchin larva [124]. Data is not available on expression of *Limpet* in other taxa in the Spiralia, but orthologs in *Drosophila* and *C. elegans* are expressed in subsets of the mesoderm [125-127]. There are no direct orthologs of *Limpet* genes in deuterostomes, but the *FHL* family in vertebrates appear to be the most closely related, having lost the PET domain characteristic of *Limpet* genes but sharing an organization of four-and-a-half LIM domains with them [128]. Members of the *FHL* family show expression in musculature and heart [129], suggesting that the ancestral role for *Limpet*/*FHL* may have been in the mesoderm.

### Morphogenesis of ventral mesoderm and a possible source of ectomesoderm

Two genes with larval expression in the ventral mesoderm, *Tt.FoxD* and *Tt.noggin*, have patterns of early expression distinct from those of the genes discussed above, which show expression in the archenteron wall. Orthologs of *Tt.FoxD* and *Tt.noggin* show comparable expression in the ventromedial mesoderm of the annelid *Platynereis* [112], a region which has been termed the ‘axochord’. In contrast to *Platynereis*, where the ventromedial region is described as being contractile [112], there is no evidence for ventromedial musculature in *T. transversa*, based on reconstructions of phalloidin-stained larvae (Figure 1 and [37]). Vertebrate *noggin* orthologs are well known for their expression in the developing mesoderm [130,131] and their role in dorsal morphogenesis [131,132]; however, additional taxonomic sampling will aid in resolving whether mesodermal *noggin* expression is conserved between deuterostomes and protostomes. Consistent with the expression of *Tt.FoxD* in both mesodermal and ectodermal tissues, *FoxD* orthologs in other taxa show expression in a range of tissues, including in the developing mesoderm of several species. In ecdysozoans, the *C. elegans* ortholog *unc-130* is expressed in the ventral mesoderm [133], while the *Drosophila* ortholog *fd59A* is restricted only to ectodermal neural tissues [134]. In the hemichordate *Saccoglossus*, the *FoxD* ortholog is expressed in the ventral mesoderm [110]; however, in echinoderms, *FoxD* expression has only been reported in ectodermal tissues [78,135]. Among chordates, *FoxD* is expressed in the notochord and somites of *Branchiostoma* [136], and the paralog *FoxD2* is expressed in the paraxial mesoderm of *Xenopus* [137] and the mouse [138]. In nearly all investigated taxa, *FoxD* orthologs also show ectodermal expression, suggesting that both mesodermal and ectodermal expression of *FoxD* may have been an ancestral trait of bilaterians, but mesodermal expression appears to have been subject to loss in multiple lineages.

Changes in the position of *Tt.noggin* and *Tt.FoxD* expression over the course of gastrulation and larval development provide fascinating clues about the morphogenesis of the mesoderm. Expression of *Tt.FoxD* at the radial gastrula stage is in the region of the archenteron wall closest to the animal pole, at the boundary with the archenteron roof. This has previously been described as the site at which endomesodermal tissue enfolds downwards to partition the endoderm and mesoderm and form a tubular gut [34]. This downward movement repositions the portion of the archenteron wall closest to the animal pole of gastrula and displaces it to the ventral side of the larva. The transition of *Tt.FoxD* expression from the animal pole boundary of the archenteron wall in the gastrula to the ventral mesoderm in the larva is consistent with these morphogenetic movements and suggests that the ventral mesoderm is specified early in development of the boundary of the endoderm and mesoderm in the archenteron. *Tt.noggin* is expressed in the lateral and posterior regions of the blastopore lip in gastrula stages but in the ventral mesoderm of larval stages. The position of these expression domains suggests that the same population of cells may express *Tt.noggin* in gastrula and larval stages. While the majority of the mesoderm is formed from the endomesoderm, which invaginates during early gastrulation to form the archenteron, the expression of *Tt.noggin* presents the possibility that cells in the lateral dorsal lip contribute to the ventral mesoderm. For both *Tt.FoxD* and *Tt.noggin*, it appears that expression in the most posterior mesodermal cells is upregulated in the early larva independent of the morphogenetic movements that place cells in the ventral mesoderm and that expression in the two distinct populations of cells coalesces by the late larval stage. A third source of tissue in the ventral mesoderm appears to be cell originally situated in the region of the archenteron wall closest to the blastopore at the vegetal pole, based on the fact that *Tt.Pax1/9* becomes localized there in the asymmetric gastrula and subsequently is expressed in the ventral mesoderm. It therefore appears that three populations of cells contribute to the ventral mesoderm of the brachiopod larva. Two endomesodermal sources, at the animal and vegetal limits of the archenteron wall, are brought together through the folding of the archenteron roof that creates the gut, while a third ectomesodermal source invaginates from the blastopore lip during closure of the blastopore.

The presence of ectomesoderm fits with current phylogenetic hypotheses which suggest that although all extant brachiopods display radial or bilateral cleavage [139], they are descended from ancestors that had spiral cleavage [19,22]. Given that the spiral cleaving taxa which are closely related to brachiopods (that is, annelids [140], nemerteans [141,142], and mollusks [143]) all develop ectomesoderm, it appears that this is plesiomorphic at least for the group Trochozoa [22] and possibly more broadly for the Spiralia in general [21], given the formation of ectomesoderm in platyhelminths [24]. Indeed, the formation of ectomesoderm in phoronids [40], which are sister to [45], or nested within [51], the brachiopod lineage, evidences that potential for an evolutionary decoupling of the specification of ectomesoderm from the stereotyped spiral cleavage program. In the future, cell lineage studies in brachiopods will help resolve whether cells expressing *Tt.noggin* in the lateral blastopore lips do indeed invaginate to form mesoderm, as our *in situ* hybridization results suggest.

### Conservation and variation in the mesodermal gene regulatory network

Previous comparisons of the genes underlying specification and differentiation of the mesoderm (and in particular musculature) have shown commonalities across bilaterian taxa, in particular between mouse and *Drosophila*, in which the most extensive developmental genetic studies have been conducted [4,6,144]. Although numerous transcription factors play roles in mesoderm specification and myogenesis in both mouse and *Drosophila*, the details of their connections and interactions in the mesodermal/myogenic gene regulatory network are divergent in these two systems [6]. Our results from expression analyses on embryonic stages in the brachiopod *T. transversa* support that a conserved set of transcription factors and nuclear proteins have roles in mesoderm specification and differentiation across the major bilaterian clades. As discussed above, the early expression of *Tt.twist* may suggest a role in the initial specification of mesoderm comparable to its function in *Drosophila* [65], despite the fact that expression of paralogs in the more closely related annelid *Capitella* is not detected until well after gastrulation [57], and in the mollusk *Patella*, the ortholog is only expressed in the ectomesoderm [64]. Later in development, broad expression of *Tt.MyoD* and *Tt.Mef2* in portions of the mesoderm fated to form muscle is consistent with conserved roles for each of these genes in myogenic specification and differentiation. On the other hand, a number of the genes described in this study, including *Tt.dachshund* and *Tt.Six1/2*, show mesodermal expression that is quite distinct from the predominantly ectodermal expression of orthologs in most other protostome taxa described to date. It may be that this represents novel aspects of mesoderm formation associated with the transition from spiral cleavage to radial cleavage in the stem lineage of brachiopods. Looking forward, sampling from a broader range of protostome taxa will likely help to distinguish how many of the genes presented here have conserved roles in mesoderm formation, versus independent recruitments with brachiopods and other taxa.

## Conclusions

The expression patterns observed suggest that several transcription factors, including *Tt.twist*, *Tt.GATA456*, *Tt.dachshund*, *Tt.NK1*, and *Tt.mPrx*, likely all play roles in specification of the mesoderm as a whole, given their expression throughout the archenteron wall during the radial gastrula stage. At the same time, localized expression of *Tt.Pax1/9*, *Tt.MyoD*, and *Tt.Six1/2* in specific regions of the archenteron wall suggests that the mesoderm is being regionalized even during the early phases of its specification. This regionalization is further reflected as larval development progresses and the expression of most genes is restricted to a subset of the larval mesoderm. The diversity of expression patterns for mesodermal genes during the development of *T. transversa* likely forms the basis for the complex musculature observed in the larva. While the majority of gene expression patterns are consistent with an endomesodermal source of mesoderm, dynamic expression of *Tt.noggin* at the blastopore suggests a previously unrecognized contribution of the ectomesoderm. Expression patterns of many genes, including *Tt.twist*, *Tt.MyoD*, and *Tt.Mef2*, are consistent with conserved roles in mesoderm differentiation and specification. Widespread mesodermal expression of *Tt.dachshund* and *Tt.Six1/2* may be an evolutionary novelty within brachiopods associated with their secondarily derived mode of radial cleavage.

## Additional files

Additional file 1:
**Summary table of mesodermal gene expression.** Compilation of mesodermal gene expression data for *T. transversa* and other bilaterian taxa, for all genes investigated in this study. Genes are organized by the developmental stage at which mesodermal expression was first detected in *T. transversa*. Additional file 2:
**GenBank accession numbers.** GenBank accession numbers are listed for *T. transversa* genes used in this study. Additional file 3:
***In situ*** **hybridization protocol.** A detailed protocol is presented for *in situ* hybridization of riboprobes in whole-mount *T. transversa* embryos, as performed for all gene expression data presented in this study. Additional file 4:
**Bayesian phylogenetic analysis of bHLH transcription factors.** Bayesian phylogenetic analysis supports orthology assignments for *Tt.MyoD*, *Tt.paraxis*, and *Tt.twist*. Additional file 5:
**Bayesian phylogenetic analysis of dachshund nuclear proteins.** Bayesian phylogenetic analysis supports orthology assignment for *Tt.dachshund*, based on placement with orthologs from the annelid *Platynereis* and the mollusk *Crassostrea*. Additional file 6:
**Bayesian phylogenetic analysis of Forkhead (Fox) transcription factors.** Bayesian phylogenetic analysis supports orthology assignments for *Tt.FoxC*, *Tt.FoxD*, and *Tt.FoxF*. Additional file 7:
**Bayesian phylogenetic analysis of MADS-box transcription factors.** Bayesian phylogenetic analysis supports orthology assignment for *Tt.MEF2*. Additional file 8:
**Bayesian phylogenetic analysis of ANTP-class homeobox transcription factors.** Bayesian phylogenetic analysis supports orthology assignment for *Tt.Mox*. Additional file 9:
**Bayesian phylogenetic analysis of NK-class homeobox transcription factors.** Bayesian phylogenetic analysis supports orthology assignment for *Tt.NK1*. Additional file 10:
**Bayesian phylogenetic analysis of Paired box (Pax) transcription factors.** Bayesian phylogenetic analysis supports orthology assignment for *Tt.Pax1/9*. Additional file 11:
**Bayesian phylogenetic analysis of sine oculis (Six) class homeobox transcription factors.** Bayesian phylogenetic analysis supports orthology assignment for *Tt.Six1/2*. Additional file 12:
**Expression patterns of** ***Tt.FoxD*** **and** ***Tt.noggin*** **during the transition for gastrula to larval stages.** All images are oriented with anterior to the left. Panels A-D and I-L are lateral views. Panels E-F and M-N are blastoporal views. Panels G-H and O-P are ventral views. For detailed descriptions of expression patterns, see text. (A-H) *Tt.FoxD* is expressed in a narrow band of cells at the border of the archenteron wall and roof in the asymmetric gastrula and transitions ventrally in the bilateral gastrula (black arrows). A second region of mesodermal expression develops in two ventrolateral posterior bands, which converge medially as the blastopore closes (white arrows). (I-P) Domains of *Tt.noggin* in the lateral regions of the blastopore lip of the asymmetric gastrula invaginate to contribute to the ventromedial mesoderm as the blastopore closes in the bilateral gastrula (white arrows). Expression in the posterior of the blastopore lip shifts to the dorsal ectoderm (black arrows), while a second domain of posterior mesodermal expression forms medially in the region that will form the pedicle lobe (white arrowheads).

## Abbreviations

BCIP: 5-bromo-4-chloro-3-indolyl phosphate
BLAST: Basic Local Alignment Search Tool
BLASTX: translated nucleotide BLAST against protein database
cDNA: complementary DNA
DIG: digoxigenin
DIG-UTP: digoxigenin-conjugated uracil triphosphate
NBT: nitro blue tetrazolium chloride
NCBI: National Center for Biotechnology Information
PCR: polymerase chain reaction
RACE: rapid amplification of cDNA ends
RT-PCR: reverse translation polymerase chain reaction
TBLASTN: protein BLAST against translated nucleotide database
