# Supplementary material for: Mesodermal gene expression during the embryonic and larval development of the articulate brachiopod Terebratalia transversa
Source: EvoDevo. 2015 Apr 11;6:10. doi: 10.1186/s13227-015-0004-8 (PMC4404124; doi:10.1186/s13227-015-0004-8)
Supplement: Additional file 1: — Summary table of mesodermal gene expression. Compilation of mesodermal gene expression data for T. transversa and other bilaterian taxa, for all genes investigated in this study. Genes are organized by the developmental stage at which mesodermal expression was first detected in T. transversa. [file 13227_2015_4_MOESM1_ESM.pdf]

Additional File 1: Mesodermal expression and function of investigated genes in other bilaterian taxa.

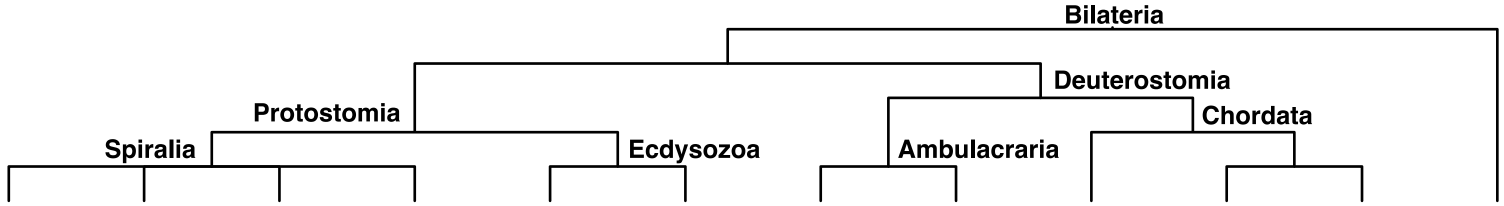

Panmesodermal in radial gastrula

Gene name	*Terebratalia*	Annelids	Mollusks	Platyhelminthes	Arthopods	Nematodes	Echinoderms	Hemichordates	Cephalochordates	Tunicates	Vertebrates	Acoels
twist	Pan-mesodermal in gastrula stages Anterior and chaetal sac mesoderm in larval stages!	Larval mesoderm [1]	Ectomesoderm [2]	Pharyngeal muscles, parenchyma [3]	Mesoderm specification [4, 5] Myogenesis [6]	Postembryonic mesoderm [7]	Primary mesenchyme cell ingression, larval skeleton, larval muscles [8]	?	Notochord, somites, anterior mesoderm [9]	Larval mesenchyme [10, 11] Juvenile mesoderm [11]	Head mesenchyme, limb bud mesenchyme, somites [12] Inhibition of myogenesis [13-15]	Muscles, gonads, neoblasts [16]
GATA456	Pan-mesodermal in radial gastrula Pedicle and chaetal sac mesoderm in larval stages (Endoderm)!	Laval trunk mesoderm [17-19] (Endoderm [18, 19])	?	Parenchyma (Endoderm) [20]	Cardiogenesis [21]	Endomesoderm [22]	Coelomic pouches [23, 24]	?	?	(Ectoderm) [25]	Cardiogenesis [26, 27] (Endoderm) [26, 27]	Muscles, gonads, neoblasts [16]
dachshund	Pan-mesodermal in gastrula stages Broadly mesodermal in larval stages (Eyes, neural, ectoderm)	Posterior mesoderm (Neural) [28]	?	(Neural) [29]	(Eyes Neural Appendages) [30-34]	(Neural) [35]	?	(Neural) [36]	Paraxial mesoderm, somites (neural) [37]	Mesenchyme (Neural) [38]	Mesenchyme, somites (eye, neural) [39, 40]	?
mPrx	Pan-mesodermal in radial gastrula Pedicle/mantle mesoderm in larval stages	?	?	?	?	?	?	Gastrula and postgastrula mesoderm [41]	?	?	Craniofacial and limb bud mesenchyme, somites, cranial skeletogenesis [42-46]	?
NK1	Pan-mesodermal in radial gastrula Pedicle mesoderm in larval stages (Ventral ectoderm)	Larval trunk mesoderm [47]	?	?	Myogenesis [48]	(Ectoderm) [49]	(Ectoderm) [50]	?	?	?	(Neural) [51-53]	?

Notes: Data on gene expression patterns in mesoderm are presented in BLACK. Data on gene function in mesoderm are presented in RED. Where relevant, or where no mesodermal expression is reported in the literature, data on gene expression and/or function are presented in parentheses. Where no data are available on the expression of a gene ortholog in a given taxon, a question mark is shown. Gene orthology groups are organized by timing or location of first detected mesodermal expression in *Terebratalia* (shown at left).

Additional File 1 continued: Mesodermal expression and function of investigated genes in other bilaterian taxa.

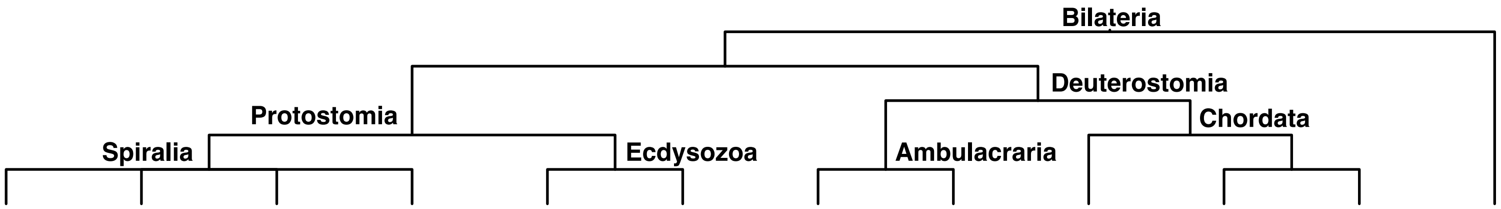

Localized mesodermal in radial gastrula

Gene name	*Terebratalia*	Annelids	Mollusks	Platyhelminthes	Arthopods	Nematodes	Echinoderms	Hemichordates	Cephalochordates	Tunicates	Vertebrates	Acoels
Pax1/9	Lateral and posterior mesoderm in gastrula stages Mantle and pedicle ventral mesoderm in larval stages	?	?	?	Body wall muscles [54]	?	?	(Pharyngeal endoderm) [55-57]	(Pharyngeal endoderm) [58]	(Pharyngeal endoderm) [55]	Sclerotome, vertebral column [59-61] (Pharyngeal endoderm) [62]	?
MyoD	Posterior mesoderm in radial gastrula Apical, mantle, and pedicle mesoderm in larval stages	?	?	?	Myogenesis [63]	Mesoderm fate specification, myogenesis [64]	Skeletogenic mesoderm, larval musculature [65]	?	Paraxial mesoderm, somatic myotomes [66]	Myogenesis [67]	Myogenesis [68]	?
Six1/2	Anterior mesoderm in radial gastrula Apical, mantle, and pedicle mesoderm in larval stages (Ectoderm)	(Eyes) [69]	?	(Eyes) [29]	(Eyes) [70]	Non-muslce mesoderm [71]	Coelomic mesoderm [65]	Gastrula and postgastrula mesoderm [41]	Invaginating endomesoderm somites [72]	(Ecotderm, endoderm) [38]	Somites, myoblast precursors, mesenchyme [73] Myogenesis [74, 75] Cranial skeletogenesis [76] Kidney development [77]	Muscles, gonads, neoblasts [16]
FoxC	Anterior mesoderm in gastrula stages Apical and ventral pedicle mesoderm in larval stages (Ectoderm)	Anterior mesoderm, posterior trunk mesoderm [78]	Anterior mesoderm, posttrochal mesoderm [78]	?	Anterior and posterior mesoderm [79]	No ortholog	Myoblasts, mesenchyme [65, 80]	Gastrula and postgastrula mesoderm [81]	Invaginating endomesoderm [82]	(Neural) [83]	Paraxial mesoderm, cardiogenesis [84-86]	Muscles, gonads, parenchyma, neoblasts [16]

Additional File 1 continued: Mesodermal expression and function of investigated genes in other bilaterian taxa.

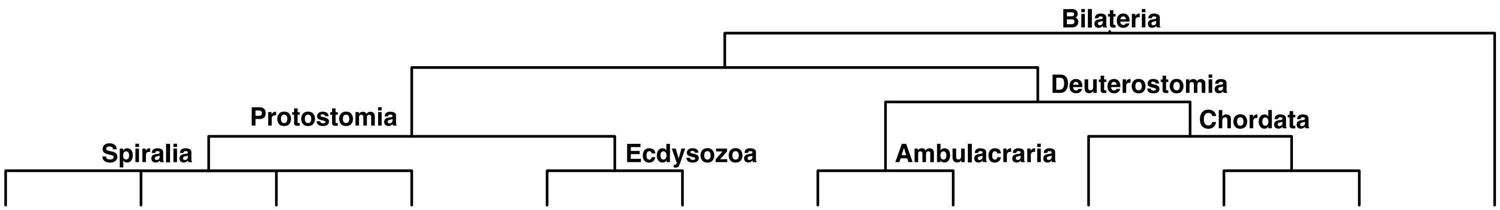

	Gene name	*Terebratalia*	Annelids	Mollusks	Platyhelminthes	Arthopods	Nematodes	Echinoderms	Hemichordates	Cephalochordates	Tunicates	Vertebrates	Acoels
Asymmetric gastrula	***Eya***	Pan-mesodermal in asymmetric gastrula Apical and pedicle mesoderm in larval stages (Ectoderm)	?	Light organ (Eyes, neural, tentacles, mantle, gills) [87]	(Eyes, neural) [29]	Myogenesis [88]	Anterior morphogenesis pharynx differentiation, gonad and vulva formation [89]	Non-skeletologenic mesoderm, coelomic mesoderm [50, 90]	(Ectoderm, pharyngeal endoderm) [57]	Invaginating endomesoderm , posterior somites, notochord [72]	Mesenchyme, trunk lateral cells [38]	Myogenesis [91] Kidney development [92]	?
Asymmetric gastrula	***FoxF***	Anterior mesoderm in asymmetric gastrula Apical and pedicle mesoderm in larval stages	Anterior mesoderm, posterior trunk mesoderm [78]	Anterior mesoderm, posttrochal mesoderm [78]	?	Visceral mesoderm [93]	Coelomocytes [94]	Non-skeletologenic mesoderm, myoblasts, [65, 80]	Gastrula and postgastrula mesoderm, heart/kidney, visceral mesoderm [81]	Ventral mesoderm [82]	Cardiogenesis [95]	Extra-embryonic mesoderm, lateral plate mesoderm [96] Splanchnic mesoderm [97, 98]	?
Larva	***Mox***	Apical/mantle and pedicle mesoderm in larval stages	Ventral mesoderm [99]	Ventral mesoderm [100]	?	Dorsal median cells 101]	No ortholog	(Neural) [102]	Ventral mesoderm [103]	Presomitic mesoderm [104]	Muscle [83]	Somitogenesis, limb musculature [105, 106]	?
Larva	***Paraxis***	Apical/mantle and pedicle mesoderm in larval stages	Ventrolateral mesoderm [99]	?	?	(Neural) [107]	No ortholog	?	?	Posterior paraxial mesoderm [108]	?	Mesoderm formation, somitogenesis, chondrogenesis, musculoskeletal patterning [109, 110]	?

Additional File 1 continued: Mesodermal expression and function of investigated genes in other bilaterian taxa.

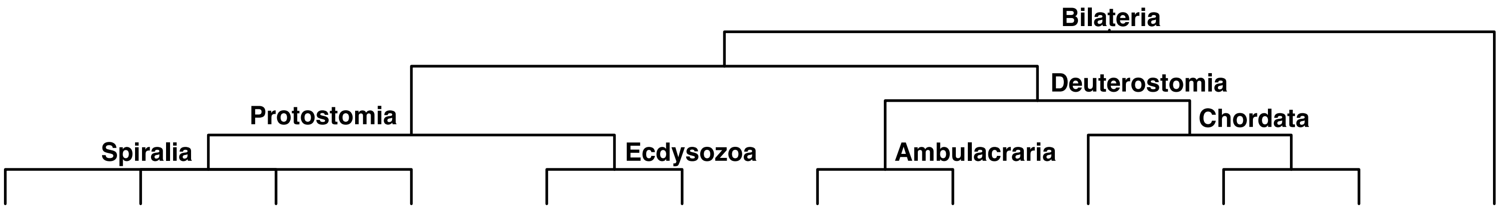

	Gene name	*Terebratalia*	Annelids	Mollusks	Platyhelminthes	Arthopods	Nematodes	Echinoderms	Hemichordates	Cephalochordates	Tunicates	Vertebrates	Acoels
Larva	***Limpet*** (*FHL* genes for vertebrates)	Apical and mantle mesoderm in early larva<br>Broadly mesodermal in late larval<br>(Ectoderm)	?	?	?	Visceral mesoderm [111]	Body wall muscle [112, 113]	?	?	?	?	Heart, somites, muscle [114-116]	?
Larva	***Mef2***	Apical and mantle mesoderm in early larva<br>Broadly mesodermal in late larval	?	?	?	Myogenesis [117, 118]	Ubiquitous<br>(not essential for myogenesis or development) [119]	Coelomic mesoderm, primary mesenchyme cells [65]	?	?	Muscle [83]	Myogenesis, cardiogenesis, chondrogenesis, neural crest [120-122]	Muscles, gonads, neoblasts [16]
Outside archenteron wall	***FoxD***	Posterior archenteron wall/roof in gastrula stages<br>Ventral mesoderm in larval stages<br>(Ectoderm)	Ventral mesoderm [99]	?	?	(Neural) [123]	Ventral muscle<br>Distal tip cell migration, axonal guidance [124]	(Ectoderm, endoderm) [50, 80]	Ventrolateral mesoderm [81]	Axial mesoderm, paraxial mesoderm, notochord, somites [125]	(Endoderm)<br>Notochord induction [126]	Somites, notochord, mesenchyme [127-129]	?
Outside archenteron wall	***noggin***	Blastopore lip in gastrula stages<br>Ventral mesoderm in larval stages<br>(Ectoderm)	Ventral mesoderm [99]	?	?	No ortholog	No ortholog	?	?	?	?	Notochord<br>Somite differentiation, neural tube formation [130-132]	?

REFERERENCES FOR SUMMARY TABLE

1. Dill KK, Thamm K, Seaver EC: **Characterization of *twist* and *snail* gene expression during mesoderm and nervous system development in the polychaete annelid *Capitella* sp. I**. *Dev Genes Evol* 2007, **217**:435–447.

2. Nederbragt AJ, Lespinet O, van Wageningen S, van Loon AE, Adoutte A, Dictus WJAG: **A lophotrochozoan *twist* gene is expressed in the ectomesoderm of the gastropod mollusk *Patella vulgata*.** *Evol Dev* 2002, **4**:334–343.

3. Martín-Durán JM, Amaya E, Romero R: **Germ layer specification and axial patterning in the embryonic development of the freshwater planarian *Schmidtea polychroa***. *Dev Biol* 2010, **340**:145-158.

4. Leptin M: ***twist* and *snail* as positive and negative regulators during *Drosophila* mesoderm development.** *Genes Dev* 1991 **5**:1568-1576.

5. Sandmann T, Girardot C, Brehme M, Tongprasit W, Stolc V, Furlong EEM: **A core transcriptional network for early mesoderm development in *Drosophila melanogaster***. *Genes Dev* 2007, **21**:436–449.

6. Baylies MK, Bate M: ***twist*: a myogenic switch in *Drosophila***. *Science* 1996, **272**:1481–1484.

7. Harfe BD, Gomes AV, Kenyon C, Liu J, Krause M, Fire A: **Analysis of a *Caenorhabditis elegans* Twist homolog identifies conserved and divergent aspects of mesodermal patterning**. *Genes Dev* 1998, **12**:2623–2635.

8. Wu S-Y, Yang Y-P, McClay DR: **Twist is an essential regulator of the skeletogenic gene regulatory network in the sea urchin embryo**. *Dev Biol* 2008, **319**:406–415.

9. Yasui K, Zhang SC, Uemura M, Aizawa S, Ueki T: **Expression of a *twist*-related gene, *Bbtwist*, during the development of a lancelet species and its relation to cephalochordate anterior structures.** *Dev Biol* 1998, **195**:49–59.

10. Imai KS: **A Twist-like bHLH gene is a downstream factor of an endogenous FGF and determines mesenchymal fate in the ascidian embryos**. *Development* 2003, **130**:4461–4472.

11. Tokuoka M, Satoh N, Satou Y: **A bHLH transcription factor gene, *Twist-like1*, is essential for the formation of mesodermal tissues of *Ciona* juveniles**. *Dev Biol* 2005, **288**:387–396.

12. Chen ZF, Behringer RR: ***twist* is required in head mesenchyme for cranial neural tube morphogenesis.** *Genes Dev* 1995, **9**:686–699.

13. Hebrok M, Wertz K, Füchtbauer E-M: ***M-twist* is an inhibitor of muscle differentiation**. *Dev Biol* 1994, **165**:537–544.

14. Spicer DB, Rhee J, Cheung WL, Lassar AB: **Inhibition of myogenic bHLH and MEF2 transcription factors by the bHLH protein Twist.** *Science* 1996, **272**:1476–1480.

15. Gong XQ: **Dermo-1, a multifunctional basic helix-loop-helix protein, represses MyoD transactivation via the HLH domain, MEF2 interaction, and chromatin deacetylation**. *Journal of Biological Chemistry* 2002, **277**:12310–12317.

16. Chiodin M, Børve A, Berezikov E, Ladurner P, Martinez P, Hejnol A: **Mesodermal gene expression in the acoel *Isodiametra* pulchra indicates a low number of mesodermal cell types and the endomesodermal origin of the gonads**. *PLoS ONE* 2013, **8**:e55499.

17. Gillis WJ, Bowerman B, Schneider SQ: **Ectoderm‐and endomesoderm‐specific GATA transcription factors in the marine annelid *Platynereis dumerilli***. *Evol Dev* 2007, **9**:39–50.

18. Boyle MJ, Seaver EC: **Developmental expression of *foxA* and *gata* genes during gut formation in the polychaete annelid, *Capitella* sp. I.** *Evol Dev* 2008, **10**:89–105.

19. Boyle MJ, Seaver EC: **Expression of *FoxA* and *GATA* transcription factors correlates with regionalized gut development in two lophotrochozoan marine worms: *Chaetopterus* (Annelida) and *Themiste lageniformis* (Sipuncula).** *Evodevo* 2010, **1**:2.

20. Martín-Durán JM, Romero R: **Evolutionary implications of morphogenesis and molecular patterning of the blind gut in the planarian *Schmidtea polychroa***. *Dev Biol* 2011, **352**:164-176.

21. Gajewski K, Fossett N, Molkentin JD, Schulz RA: **The zinc finger proteins Pannier and GATA4 function as cardiogenic factors in *Drosophila*.** *Development* 1999, **126**:5679–5688.

22. Maduro MF, Meneghini MD, Bowerman B, Broitman-Maduro G, Rothman JH: **Restriction of mesendoderm to a single blastomere by the combined action of SKN-1 and a GSK-3beta homolog is mediated by MED-1 and -2 in *C. elegans*.** *Mol Cell* 2001, **7**:475–485.

23. Hinman VF, Davidson EH: **Expression of a gene encoding a Gata transcription factor during embryogenesis of the starfish *Asterina miniata***. *Gene Expr Patterns* 2003, **3**:419–422.

24. Lee PY, Davidson EH: **Expression of *Spgatae*, the *Strongylocentrotus***

***purpuratus* ortholog of vertebrate GATA4/5/6 factors**. *Gene Expression Patterns* 2004, **5**:161–165.

25. Rothbächer U, Bertrand V, Lamy C, Lemaire P: **A combinatorial code of maternal GATA, Ets and beta-catenin-TCF transcription factors specifies and patterns the early ascidian ectoderm.** *Development* 2007, **134**:4023–4032.

26. Kuo CT, Morrisey EE, Anandappa R, Sigrist K, Lu MM, Parmacek MS, Soudais C, Leiden JM: **GATA4 transcription factor is required for ventral morphogenesis and heart tube formation.** *Genes Dev* 1997, **11**:1048–1060.

27. Molkentin JD, Lin Q, Duncan SA, Olson EN: **Requirement of the transcription factor GATA4 for heart tube formation and ventral morphogenesis.** *Genes Dev* 1997, **11**:1061–1072.

28. Winchell CJ, Valencia JE, Jacobs DK: **Expression of *Distal-less*, *dachshund*, and *optomotor blind* in *Neanthes arenaceodentata* (Annelida, Nereididae) does not support homology of appendage-forming mechanisms across the Bilateria**. *Dev Genes Evol* 2010, **220**:275–295.

29. Martín-Durán JM, Monjo F, Romero R: **Morphological and molecular development of the eyes during embryogenesis of the freshwater planarian *Schmidtea polychroa***. *Dev Genes Evol* 2012, **222**:45–54.

30. Mardon G, Solomon NM, Rubin GM: ***dachshund* encodes a nuclear protein required for normal eye and leg development in *Drosophila***. *Development* 1994, **120**:3473-3486.

31. Prpic N-M, Wigand B, Damen W, Klingler M: **Expression of *dachshund* in wild-type and *Distal-less* mutant *Tribolium* corroborates serial homologies in insect appendages**. *Dev Genes Evol* 2001, **211**:467–477.

32. Prpic N-M, Tautz D: **The expression of the proximodistal axis patterning genes *Distal-less* and *dachshund* in the appendages of *Glomeris marginata* (Myriapoda: Diplopoda) suggests a special role of these genes in patterning the head appendages**. *Dev Biol* 2003, **260**:97–112.

33. Inoue Y, Miyawaki K, Terasawa T, Matsushima K, Shinmyo Y, Niwa N, Mito T, Ohuchi H, Noji S: **Expression patterns of *dachshund* during head development of *Gryllus bimaculatus* (cricket)**. *Gene Expr Patterns* 2004, **4**:725–731.

34. Sharma PP, Schwager EE, Extavour CG: **Evolution of the chelicera: a *dachshund* domain is retained in the deutocerebral appendage of Opiliones (Arthropoda, Chelicerata)**. *Evol Dev* 2012.

35. Colosimo ME, Brown A, Mukhopadhyay S, Gabel C, Lanjuin AE, Samuel

ADT, Sengupta P: **Identification of hermosensory and olfactory neuron-specific genes via expression profiling of single neuron types**. *Curr Biol* 2004, **14**:2245–2251.

36. Lemons D, Fritzenwanker JH, Gerhart J, Lowe CJ, McGinnis W: **Co-option of an anteroposterior head axis patterning system for proximodistal patterning of appendages in early bilaterian evolution**. *Dev Biol* 2010, **344**:358–362.

37. Candiani S, Kreslova J, Benes V, Oliveri D, Castagnola P, Pestarino M, Kozmik Z: **Cloning and developmental expression of amphioxus *Dachschund***. *Gene Expr Patterns* 2003, **3**:65–69.

38. Mazet F, Hutt JA, Milloz J, Millard J, Graham A, Shimeld SM: **Molecular evidence from *Ciona intestinalis* for the evolutionary origin of vertebrate sensory placodes**. *Dev Biol* 2005, **282**:494–508.

39. Caubit X, Thangarajah R, Theil T, Wirth J, Nothwang HG, Rüther U, Krauss S: **Mouse Dac, a novel nuclear factor with homology to *Drosophila* dachshund shows a dynamic expression in the neural crest, the eye, the neocortex, and the limb bud.** *Dev Dyn* 1999, **214**:66–80.

40. Hammond KL, Hill RE, Whitfield TT: **Isolation of three zebrafish *dachshund* homologues and their expression in sensory organs, the central nervous system and pectoral fin buds**. *Mech Dev* 2002, **112**:183-189.

41. Green SA, Norris RP, Terasaki M, Lowe CJ: **FGF signaling induces mesoderm in the hemichordate *Saccoglossus kowalevskii***. *Development* 2013, **140**:1024–1033.

42. Opstelten D, Vogels R, Robert B, Kalkhoven E: **The mouse homeobox gene, *S8*, is expressed during embryogenesis predominantly in mesenchyme**. *Mech Dev* 1991, **34**:29-41.

43. Cserjesi P, Lilly B, Bryson L, Wang Y, Sassoon DA, Olson EN: **MHox: a mesodermally restricted homeodomain protein that binds an essential site in the muscle creatine kinase enhancer**. *Development* 1992, **115**:1087-1101.

44. Leussink B, Brouwer A, Khattabi El M, Poelmann RE, Gittenberger-de Groot AC, Meijlink F: **Expression patterns of the paired-related homeobox genes *MHox*/*Prx1* and *S8*/*Prx2* suggest roles in development of the heart and the forebrain**. *Mech Dev* 1995, **52**:51–64.

45. Berge ten D, Brouwer A, Korving J, Martin JF, Meijlink F: ***Prx1* and *Prx2* in skeletogenesis: roles in the craniofacial region, inner ear and limbs.** *Development* 1998, **125**:3831–3842.

46. Lu MF, Cheng HT, Kern MJ, Potter SS, Tran B, Diekwisch TG, Martin JF:

prx-1 **functions cooperatively with another paired-related homeobox gene,** ***prx-2*****, to maintain cell fates within the craniofacial mesenchyme.** *Development* 1999, **126**:495–504.

47. Saudemont A, Dray N, Hudry B, Le Gouar M, Vervoort M, Balavoine G: **Complementary striped expression patterns of *NK* homeobox genes during segment formation in the annelid *Platynereis***. *Dev Biol* 2008, **317**:430–443.

48. Knirr S, Azpiazu N, Frasch M: **The role of the NK-homeobox gene slouch (*S59*) in somatic muscle patterning**. *Development* 1999,**126**:4525-4535.

49. Reece-Hoyes JS, Shingles J, Dupuy D, Grove CA, Walhout AJM, Vidal M, Hope IA: **Insight into transcription factor gene duplication from *Caenorhabditis elegans* Promoterome-driven expression patterns.** *BMC Genomics* 2007, **8**:27.

50. Yankura KA, Martik ML, Jennings CK, Hinman VF: **Uncoupling of complex regulatory patterning during evolution of larval development in echinoderms.** *BMC Biology* 2010, **8**:143.

51. Schubert FR, Fainsod A, Gruenbaum Y, Gruss P: **Expression of the novel murine homeobox gene *Sax-1* in the developing nervous system.** *Mech Dev* 1995, **51**:99–114.

52. Simon R, Lufkin T: **Postnatal lethality in mice lacking the *Sax2* homeobox gene homologous to *Drosophila S59/slouch*: evidence for positive and negative autoregulation**. *Mol Cell Biol* 2003, **23**:9046–9060.

53. Bae Y-K, Shimizu T, Muraoka O, Yabe T, Hirata T, Nojima H, Hirano T, Hibi M: **Expression of *sax1/nkx1.2* and *sax2/nkx1.1* in zebrafish**. *Gene Expr Patterns* 2004, **4**:481–486.

54. Duan H, Zhang C, Chen J, Sink H, Frei E, Noll M: **A key role of *Pox meso* in somatic myogenesis of *Drosophila*.** *Development* 2007, **134**:3985–3997.

55. Ogasawara M, Wada H, Peters H, Satoh N: **Developmental expression of *Pax1/9* genes in urochordate and hemichordate gills: insight into function and evolution of the pharyngeal epithelium**. *Development* 1999, **126**:2539:2550.

56. Lowe CJ, Wu M, Salic A, Evans L, Lander E, Stange-Thomann N, Gruber CE, Gerhart J, Kirschner M: **Anteroposterior patterning in hemichordates and the origins of the chordate nervous system.** *Cell* 2003, **113**:853–865.

57. Gillis JA, Fritzenwanker JH, Lowe CJ: **A stem-deuterostome origin of the vertebrate pharyngeal transcriptional network**. *Proc R Soc Lond B Biol* 2011, **279**:237–246.

58. Holland ND, Holland LZ, Kozmik Z: **An amphioxus Pax gene, *AmphiPax-1*, expressed in embryonic endoderm, but not in mesoderm: implications for the evolution of class I paired box genes.** *Mol Mar Biol Biotechnol* 1995, **4**:206–214.

59. Wallin J, Wilting J, Koseki H, Fritsch R, Christ B, Balling R: **The role of *Pax-1* in axial skeleton development**. 1994, Development, **120**:1109-1121.

60. Peters H, Wilm B, Sakai N, Imai K, Maas R, Balling R: **Pax1 and Pax9 synergistically regulate vertebral column development**. *Development* 1999, **126**:5399-5408.

61. Mise T, Iijima M, Inohaya K, Kudo A, Wada H: **Function of *Pax1* and *Pax9* in the sclerotome of medaka fish.** *Genesis* 2008, **46**:185–192.

62. Ogasawara M, Shigetani Y, Hirano S, Satoh N, Kuratani S: ***Pax1*/*Pax9*-related genes in an agnathan vertebrate, *Lampetra* japonica: expression pattern of *LjPax9* implies sequential evolutionary events toward the gnathostome body plan**. *Dev Biol* 2000, **223**:399–410.

63. Balagopalan L, Keller CA, Abmayr SM: **Loss-of-function mutations reveal that the *Drosophila nautilus* gene is not essential for embryonic myogenesis or viability**. *Dev Biol* 2001, **231**:374–382.

64. Harfe BD, Branda CS, Krause M, Stern MJ, Fire A: **MyoD and the specification of muscle and non-muscle fates during postembryonic development of the *C. elegans* mesoderm.** *Development* 1998, **125**:2479–2488.

65. Andrikou C, Iovene E, Rizzo F, Oliveri P, Arnone MI: **Myogenesis in the sea urchin embryo: the molecular fingerprint of the myoblast precursors.** *Evodevo* 2013, **4**:33.

66. Schubert M, Meulemans D, Bronner-Fraser M: **Differential mesodermal expression of two amphioxus *MyoD* family members (*AmphiMRF1* and *AmphiMRF2*)**. *Gene Expr Patterns* 2003, **3**:199-202.

67. Meedel TH, Chang P, Yasuo H: **Muscle development in *Ciona intestinalis* requires the b-HLH myogenic regulatory factor gene *Ci-MRF***. *Dev Biol* 2007, **302**:333–344.

68. Rudnicki MA, Schnegelsberg P, Stead RH, Braun T: **MyoD or Myf-5 is required for the formation of skeletal muscle**. *Cell* 1993, **75**:1351–1359.

69. Arendt D, Tessmar K, de Campos-Baptista M-IM, Dorresteijn A, Wittbrodt J: **Development of pigment-cup eyes in the polychaete *Platynereis dumerilii* and evolutionary conservation of larval eyes in Bilateria**. *Development* 2002, **129**:1143-1154.

70. Cheyette BN, Green PJ, Martin K, Garren H, Hartenstein V, Zipursky SL: **The *Drosophila sine oculis* locus encodes a homeodomain-containing protein required for the development of the entire visual system.** *Neuron* 1994, **12**:977–996.

71. Amin NM, Lim SE, Shi H, Chan TL, Liu J: **A conserved Six–Eya cassette acts downstream of Wnt signaling to direct non-myogenic versus myogenic fates in the *C. elegans* postembryonic mesoderm**. *Dev Biol* 2009, **331**:350-360.

72. Kozmik Z, Holland ND, Kreslova J, Oliveri D: **Pax–Six–Eya–Dach network during amphioxus development: Conservation in vitro but context specificity in vivo**. *Dev Biol* 2007, **306**:143-159.

73. Oliver G, Wehr R, Jenkins NA, Copeland NG, Cheyette BN, Hartenstein V, Zipursky SL, Gruss P: **Homeobox genes and connective tissue patterning.** *Development* 1995, **121**:693–705.

74. Grifone R, Demignon J, Houbron C, Souil E, Niro C, Seller MJ, Hamard G, Maire P: **Six1 and Six4 homeoproteins are required for Pax3 and Mrf expression during myogenesis in the mouse embryo.** *Development* 2005, **132**:2235–2249.

75. Niro C, Demignon J, Vincent S, Liu Y, Giordani J: **Six1 and Six4 gene expression is necessary to activate the fast-type muscle gene program in the mouse primary myotome**. *Dev Biol* 2010, **338**:168-182.

76. He G, Tavella S, Hanley KP, Self M, Oliver G, Grifone R, Hanley N, Ward C, Bobola N: **Inactivation of *Six2* in mouse identifies a novel genetic mechanism controlling development and growth of the cranial base**. *Dev Biol* 2010, **344**:720–730.

77. Kobayashi A, Valerius MT, Mugford JW, Carroll TJ, Self M, Oliver G, McMahon AP: **Six2 defines and regulates a multipotent self-renewing nephron progenitor population throughout mammalian kidney development**. *Cell Stem Cell* 2008, **3**:169–181.

78. Shimeld SM, Boyle MJ, Brunet T, Luke GN, Seaver EC: **Clustered Fox genes in lophotrochozoans and the evolution of the bilaterian Fox gene cluster**. *Dev Biol* 2010, **340**:234–248.

79. Häcker U, Kaufmann E, Hartmann C, Jürgens G, Knöchel W, Jäckle H: **The *Drosophila fork head* domain protein crocodile is required for the establishment of head structures.** *EMBO J* 1995, **14**:5306–5317.

80. Tu Q, Brown CT, Davidson EH, Oliveri P: **Sea urchin Forkhead gene family: Phylogeny and embryonic expression**. *Dev Biol* 2006, **300**:49-62.

81. Fritzenwanker JH, Gerhart J, Freeman RM, Lowe CJ: **The Fox/Forkhead transcription factor family of the hemichordate *Saccoglossus kowalevskii***. *Evodevo* 2014, **5**:17.

82. Mazet F, Amemiya CT, Shimeld SM: **An ancient Fox gene cluster in bilaterian animals.** *Curr Biol* 2006, **16**:R314–6.

83. Imai KS, Hino K, Yagi K, Satoh N, Satou Y: **Gene expression profiles of transcription factors and signaling molecules in the ascidian embryo: towards a comprehensive understanding of gene networks.** *Development* 2004, **131**:4047–4058.

84. Winnier GE, Hargett L, Hogan BL: **The winged helix transcription factor *MFH1* is required for proliferation and patterning of paraxial mesoderm in the mouse embryo.** *Genes Dev* 1997, **11**:926–940.

85. Kume T, Jiang H, Topczewska JM, Hogan BL: **The murine winged helix transcription factors, Foxc1 and Foxc2, are both required for cardiovascular development and somitogenesis.** *Genes Dev* 2001, **15**:2470–2482.

86. Wilm B, James RG, Schultheiss TM, Hogan BLM: **The forkhead genes, *Foxc1* and *Foxc2*, regulate paraxial versus intermediate mesoderm cell fate**. *Dev Biol* 2004, **271**:176–189.

87. Peyer SM, Pankey MS, Oakley TH, McFall-Ngai MJ: **Eye-specification genes in the bacterial light organ of the bobtail squid *Euprymna scolopes*, and their expression in response to symbiont cues.** *Mech Dev* 2014, **131**:111–126.

88. Liu Y-H, Jakobsen JS, Valentin G, Amarantos I, Gilmour DT, Furlong EEM: **A systematic analysis of Tinman function reveals Eya and JAK-STAT signaling as essential regulators of muscle development**. *Dev Cell* 2009, **16**:280–291.

89. Furuya M, Qadota H, Chisholm AD, Sugimoto A: **The *C. elegans* eyes absent ortholog EYA-1 is required for tissue differentiation and plays partially redundant roles with PAX-6**. *Dev Biol* 2005, **286**:452–463.

90. Materna SC, Ransick A, Li E, Davidson EH: **Diversification of oral and aboral mesodermal regulatory states in pregastrular sea urchin embryos**. *Dev Biol* 2013, **375**:92–104.

91. Heanue TA, Reshef R, Davis RJ, Mardon G, Oliver G, Tomarev S, Lassar AB, Tabin CJ: **Synergistic regulation of vertebrate muscle development by *Dach2*, *Eya2*, and *Six1*, homologs of genes required for *Drosophila* eye formation**. *Genes Dev* 1999, **13**:3231-3243.

92. Xu PX, Adams J, Peters H, Brown MC, Heaney S, Maas R: ***Eya1*-deficient mice lack ears and kidneys and show abnormal apoptosis of organ primordia.** *Nat Genet* 1999, **23**:113–117.

93. Zaffran S, Küchler A, Lee HH, Frasch M: ***biniou* (*FoxF*), a central component in a regulatory network controlling visceral mesoderm development and midgut morphogenesis in *Drosophila*.** *Genes Dev* 2001, **15**:2900–2915.

94. Amin NM, Shi H, Liu J: **The FoxF/FoxC factor LET-381 directly regulates both cell fate specification and cell differentiation in *C. elegans* mesoderm development**. *Development* 2010, **137**:1451–1460.

95. Beh J, Shi W, Levine M, Davidson B, Christiaen L: ***FoxF* is essential for FGF-induced migration of heart progenitor cells in the ascidian *Ciona intestinalis***. *Development* 2007, **134**:3297–3305.

96. Mahlapuu M, Ormestad M, Enerback S, Carlsson P: **The forkhead transcription factor Foxf1 is required for differentiation of extra-embryonic and lateral plate mesoderm**. *Development* 2001, **128**:155-166.

97. Ormestad M, Astorga J, Carlsson P: **Differences in the embryonic expression patterns of mouse *Foxf1* and *-2* match their distinct mutant phenotypes.** *Dev Dyn* 2004, **229**:328–333.

98. Ormestad M: ***Foxf1* and *Foxf2* control murine gut development by limiting mesenchymal Wnt signaling and promoting extracellular matrix production**. *Development* 2006, **133**:833–843.

99. Lauri A, Brunet T, Handberg-Thorsager M, Fischer AHL, Simakov O, Steinmetz PRH, Tomer R, Keller PJ, Arendt D: **Development of the annelid axochord: Insights into notochord evolution**. *Science* 2014, **345**:1365–1368.

100. Hinman VF, Degnan BM: ***Mox* homeobox expression in muscle lineage of the gastropod *Haliotis asinina*: evidence for a conserved role in bilaterian myogenesis.** *Dev Genes Evol* 2002, **212**:141–144.

101. Chiang C, Patel NH, Young KE, Beachy PA: **The novel homeodomain gene *buttonless* specifies differentiation and axonal guidance functions of *Drosophila* dorsal median cells**. *Development* 1994, **120**:3581-3593.

102. Poustka AJ, Kühn A, Groth D, Weise V, Yaguchi S, Burke RD, Herwig R, Lehrach H, Panopoulou G: **A global view of gene expression in lithium and zinc treated sea urchin embryos: new components of gene regulatory networks**. *Genome Biol* 2007, **8**:R85.

103. Lowe CJ, Terasaki M, Wu M, Freeman RM, Runft L, Kwan K, Haigo S, Aronowicz J, Lander E, Gruber C, Smith M, Kirschner M, Gerhart J:

Dorsoventral patterning in hemichordates: insights into early chordate evolution. *PLoS Biol* 2006, **4**:e291.

104. Minguillón C, Garcia-Fernàndez J: **The single amphioxus Mox gene: insights into the functional evolution of mox genes, somites, and the asymmetry of amphioxus somitogenesis**. *Dev Biol* 2002, **246**:455-465.

105. Mankoo BS, Collins NS, Ashby P, Grigorieva E, Pevny LH, Candia A, Wright CV, Rigby PW, Pachnis V: ***Mox2* is a component of the genetic hierarchy controlling limb muscle development.** *Nature* 1999, **400**:69–73.

106. Mankoo BS: **The concerted action of Meox homeobox genes is required upstream of genetic pathways essential for the formation, patterning and differentiation of somites**. *Development* 2003, **130**:4655–4664.

107. Peyrefitte S, Kahn D, Haenlin M: **New members of the *Drosophila* Myc transcription factor subfamily revealed by a genome-wide examination for basic helix-loop-helix genes.** *Mech Dev* 2001, **104**:99–104.

108. Beaster-Jones L, Kaltenbach SL, Koop D, Yuan S, Chastain R, Holland LZ: **Expression of somite segmentation genes in amphioxus: a clock without a wavefront?** *Dev Genes Evol* 2008, **218**:599–611.

109. Burgess R, Rawls A, Brown D, Bradley A, Olson EN: **Requirement of the *paraxis* gene for somite formation and musculoskeletal patterning.** *Nature* 1996, **384**:570–573.

110. Brown D, Wagner D, Li X, Richardson JA, Olson EN: **Dual role of the basic helix-loop-helix transcription factor scleraxis in mesoderm formation and chondrogenesis during mouse embryogenesis.** *Development* 1999, **126**:4317–4329.

111. Tomancak P, Berman BP, Beaton A, Weiszmann R, Kwan E, Hartenstein V, Celniker SE, Rubin GM: **Global analysis of patterns of gene expression during *Drosophila* embryogenesis.** *Genome Biol* 2007, **8**:R145.

112. Qadota H, Mercer KB, Miller RK, Kaibuchi K, Benian GM: **Two LIM domain proteins and UNC-96 link UNC-97/PINCH to myosin thick filaments in *Caenorhabditis elegans* muscle**. *Mol Biol Cell* 2007, **18**:4317–4326.

113. Xiong G, Qadota H, Mercer KB, McGaha LA: **A LIM-9 (FHL) / SCPL-1 (SCP) complex interacts with the C-terminal protein kinase regions of UNC-89 (Obscurin) in *Caenorhabditis elegans* muscle**. *J Mol Biol* 2009, **386**:976-988.

114. Morgan MJ, Madgwick AJ: **The LIM proteins FHL1 and FHL3 are expressed differently in skeletal muscle.** *Biochem Biophys Res Commun* 1999, **255**:245–250.

115. Chu P-H, Ruiz-Lozano P, Zhou Q, Cai C, Chen J: **Expression patterns of FHL/SLIM family members suggest important functional roles in skeletal muscle and cardiovascular system**. *Mech Dev* 2000, **95**:259–265.

116. Martin B, Schneider R, Janetzky S, Waibler Z, Pandur P, Kühl M, Behrens J, Mark von der K, Starzinski-Powitz A, Wixler V: **The LIM-only protein FHL2 interacts with beta-catenin and promotes differentiation of mouse myoblasts.** *J Cell Biol* 2002, **159**:113–122.

117. Bour BA, O'Brien MA, Lockwood WL, Goldstein ES, Bodmer R, Taghert PH, Abmayr SM, Nguyen HT: ***Drosophila* MEF2, a transcription factor that is essential for myogenesis.** *Genes Dev* 1995, **9**:730–741.

118. Ranganayakulu G, Zhao B, Dokidis A, Molkentin JD: **A series of mutations in the D-MEF2 transcription factor reveal multiple functions in larval and adult myogenesis in *Drosophila***. *Dev Biol* 1995, **171**:169-181.

119. Dichoso D, Brodigan T, Chwoe KY, Lee JS, Llacer R, Park M, Corsi AK, Kostas SA, Fire A, Ahnn J, Krause M: **The MADS-Box factor CeMEF2 is not essential for *Caenorhabditis elegans* myogenesis and development**. *Dev Biol* 2000, **223**:431–440.

120. Molkentin JD, Black BL, Martin JF, Olson EN: **Cooperative activation of muscle gene expression by MEF2 and myogenic bHLH proteins**. *Cell* 1995, **83**:1125–1136.

121. Potthoff MJ, Arnold MA, McAnally J, Richardson JA, Bassel-Duby R, Olson EN: **Regulation of skeletal muscle sarcomere integrity and postnatal muscle function by *Mef2c*.** *Mol Cell Biol* 2007, **27**:8143–8151.

122. Potthoff MJ, Olson EN: **MEF2: a central regulator of diverse developmental programs.** *Development* 2007, **134**:4131–4140.

123. Lee H-H, Frasch M: **Survey of forkhead domain encoding genes in the *Drosophila* genome: Classification and embryonic expression patterns.** *Dev Dyn* 2004, **229**:357–366.

124. Nash B, Colavita A, Zheng H, Roy PJ, Culotti JG: **The forkhead transcription factor UNC-130 is required for the graded spatial expression of the UNC-129 TGF-beta guidance factor in *C. elegans*.** *Genes Dev* 2000, **14**:2486–2500.

125. Yu JK, Holland ND, Holland LZ: **An amphioxus winged helix/forkhead gene, *AmphiFoxD*: insights into vertebrate neural crest evolution.** *Dev Dyn* 2002, **225**:289–297.

126. Imai KS, Satoh N, Satou Y: **An essential role of a *FoxD* gene in notochord induction in *Ciona* embryos**. *Development* 2002, **129**:3441–3453.

127. Hatini V, Huh SO, Herzlinger D, Soares VC, Lai E: **Essential role of stromal mesenchyme in kidney morphogenesis revealed by targeted disruption of Winged Helix transcription factor *BF-2*.** *Genes Dev* 1996, **10**:1467–1478.

128. Gómez-Skarmeta JL, la Calle-Mustienes de E, Modolell J, Mayor R: ***Xenopus brain factor-2* controls mesoderm, forebrain and neural crest development.** *Mech Dev* 1999, **80**:15–27.

129. Steiner AB, Engleka MJ, Lu Q, Piwarzyk EC, Yaklichkin S, Lefebvre JL, Walters JW, Pineda-Salgado L, Labosky PA, Kessler DS: **FoxD3 regulation of Nodal in the Spemann organizer is essential for *Xenopus* dorsal mesoderm development**. *Development* 2006, **133**:4827–4838.

130. Smith WC, Harland RM: **Expression cloning of noggin, a new dorsalizing factor localized to the Spemann organizer in *Xenopus* embryos.** *Cell* 1992, **70**:829–840.

131. Smith WC, Knecht AK, Wu M, Harland RM: **Secreted noggin protein mimics the Spemann organizer in dorsalizing *Xenopus* mesoderm.** *Nature* 1993, **361**:547–549.

132. McMahon JA, Takada S, Zimmerman LB, Fan CM, Harland RM, McMahon AP: **Noggin-mediated antagonism of BMP signaling is required for growth and patterning of the neural tube and somite**. *Genes Dev* 1998, **12**:1438–1452.
