# Supplementary material for: Mesodermal gene expression during the embryonic and larval development of the articulate brachiopod Terebratalia transversa
Source: EvoDevo. 2015 Apr 11;6:10. doi: 10.1186/s13227-015-0004-8 (PMC4404124; doi:10.1186/s13227-015-0004-8)
Supplement: Additional file 2: — GenBank accession numbers. GenBank accession numbers are listed for T. transversa genes used in this study. [file 13227_2015_4_MOESM2_ESM.pdf]

Gene	GenBank accession
Tt.dachshund	KP168451
Tt.eya	KP168452
Tt.FoxC	KP168453
Tt.FoxD	KP168454
Tt.FoxF	KP168455
Tt.GATA456	KP168456
Tt.Limpet	KP168457
Tt.MEF2	KP168458
Tt.mPrx	KP168459
Tt.Mox	KP168460
Tt.MyoD	KP168461
Tt.NK1	KP168462
Tt.noggin	KP168463
Tt.paraxis	KP168464
Tt.Pax1/9	KP168465
Tt.Six1/2	KP168466
Tt.tropomyosin	DQ493898
Tt.twist	KP168467
