# Supplementary material for: Mesodermal gene expression during the embryonic and larval development of the articulate brachiopod Terebratalia transversa
Source: EvoDevo. 2015 Apr 11;6:10. doi: 10.1186/s13227-015-0004-8 (PMC4404124; doi:10.1186/s13227-015-0004-8)
Supplement: Additional file 3: — In situ hybridization protocol. A detailed protocol is presented for in situ hybridization of riboprobes in whole-mount T. transversa embryos, as performed for all gene expression data presented in this study. [file 13227_2015_4_MOESM3_ESM.pdf]

Terebratalia in-situ hybridization protocol

-DAY 1-

Pretreatment

- Transfer embryos to a 24 well dish and use 500µl for each wash.
  Rehydrate through: 60% MeOH/40% PTw
  30% MeOH/70% PTw
  4 x PTw washes
- Digest with Proteinase-K (0.01 mg/ml in PTw) for 10 minutes (no shaker) *[5 µl per 10 ml]*
- Stop digestion with 2 (PTw + 2 mg/ml glycine made up fresh) washes. *[0.1 g per 50 ml]*
- Wash 1x with 1% triethanolamine in PTw (made up fresh),
- Wash 1x with 1% triethanolamine in Ptw, plus1.5 µl acetic anhydride. (The acetic anhydride will not immediately mix with the triethanolamine. Swirl and rock to ensure it mixes).
- Wash 1x with 1% triethanolamine in Ptw, plus 3 µl acetic anhydride.
- Wash 2 x in PTw
- Refix in 3.7% formaldehyde in PTw for 1 hour at RT. *[1 ml per 10 ml]*
- Wash 5 x in PTw

Prehybe

- Remove as much liquid as possible, add 500 µl hybe buffer - incubate for 10 minutes at RT.
- Remove liquid - add 500 µl hybe buffer. Place at hybe temp overnight (62ºC)

Hybe

- Dilute probe to a final concentration of 10-0.05 ng/µl (usually 1.0 ng/µl) in hybe solution (dig-labeled probe should be stored as a 50 ng/µl stock in hybe buffer at -20 degrees). Denature probe at 80-90°C max for 10 minutes. Remove prehybe and add probe to each well. Hybridize over the weekend.

-DAY 2-

- Remove hybe (can be reused 4-5 times)
- Wash 1 x for 10 minutes and 1 x for 40 minutes with hybe buffer at hybe temp. (Do not forget to prewarm hybe buffer)

Washes (for non-hybe washes, use 900 µl per wash)

- 30 min in 75% hybe + 25% 2X SSC (pH 7.0) at hybe temp
- 30 min in 50% hybe + 50% 2X SSC at hybe temp
- 30 min in 25% hybe + 75% 2X SSC at hybe temp
- 30 min in 100% 2X SSC at hybe temp

- gloves no longer required -

- 3 x 20 min in 0.05X SSC at hybe temp
- 10 min in 75% 0.05X SSC + 25% PTw at RT
- 10 min in 50% 0.05X SSC + 50% PTw at RT
- 10 min in 25% 0.05X SSC + 75% PTw at RT
- 10 min in 100% PTw at RT

Visualization of Probe

- Wash 5 x with PBT at RT
- Block in Boehringer-Mannheim Blocking buffer (diluted to 1x with maleic acid buffer) 1 hr at RT on rocker (or overnight at 4°C).
- Incubate with Boehringer-Mannheim anti-Dig/AP (diluted in blocking buffer to 1:5000) at 4°C overnight on rocker. (Can also incubate 1-4 hrs at room temp)

-DAY3-

- Wash 10x (or more) for 15-30 minutes in PBT.
- Wash 1x for 10 minutes in AP Buffer without $MgCl_2$
- Wash 2x for 10 minutes in AP buffer
- Develop in AP substrate solution (make fresh) at RT in dark. Monitor color development. (Can also develop slower at 4 degrees)
- Stop reaction by washing 5 x with PTw. Mount in 70% glycerol in PTw.

SOLUTIONS:

**Hybe Buffer** (40 mL)	ADD	[FINAL]
formamide	20 mL	50%
20x SSC (pH 4.5)	10 mL	5x
20 mg/mL heparin	0.1 mL	50 µg/mL
*20% Tween-20	0.2 mL	0.1%
20% SDS	2.0 mL	1.0%
10 mg/mL SS DNA	0.2 mL	100 µg/mL
dH_2O	7.8 mL	

10x PBS = 18.6 mM NaH_2PO_4 (2.56 g NaH_2PO_4-H_2O per liter dH_2O)
84.1 mM Na_2HPO_4 (11.94 g Na_2HPO_4-H_2O per liter dH_2O)
1,750 mM NaCl (102.2 g NaCl per liter dH_2O)

Mix phosphates in about 800 mL of dH_2O for a 1.0 L volume. Check pH. It should be 7.4 ± 0.4. If more than 0.4 off, start over. Otherwise adjust pH to 7.4 with NaOH or HCl. Add the NaCl and rest of dH_2O.

PTw = 1x PBS + 0.1% Tween-20 detergent
(100 ml 10x PBS, 895 ml dH_2O, DEPC-treat/autoclave; when cool, add 5 ml 20% Tween)

PBT = 1x PBS + 0.2% Triton X-100 + 0.1% BSA (store at 4 degrees C)
(to 990 mls 1x PBS, add 10 mls 20% Triton X-100, then add 1g BSA and filter sterilize)

20x SSC = 0.3 M Na citrate + 3 M NaCl
(for 1 L, add 175.3 g NaCl + 88.2 g Na citrate, pH to 4.5 or 7.0, and autoclave)

**Alkaline Phosphatase buffer (50mL)**		ADD	[FINAL]
1 M	NaCl	5.0 ml	100 mM
1 M	$MgCl_2$	2.5 ml	50 mM
1 M	Tris, pH 9.5	5.0 ml	100 mM
20%	Tween-20	1.25 ml	0.5%
	dH_2O	36.25 ml	

(prepare just prior to use. The solution will become cloudy after a few hours and will no longer work for the enzymatic reaction)

AP Substrate Solution

To AP buffer, add 3.3 µl/ml NBT (stock: 50 mg/ml in 70% dimethyl formamide:30% water) and then 3.3 µl/ml BCIP (stock: 50 mg/ml in dimethyl formamide). Keep this solution dark.

Notes:

Use RNAse-free equipment and solutions (DEPC treated) through hybridization step. All washes are 5 min. at RT on rocker table unless otherwise stated.

Activity of Proteinase K can vary between vendors, and between lots. Each new lot received should be tested empirically to optimize results.

Hybridization temperature can range from 55 to 65 degrees, depending on probe and stringency needed. Normally prehybe overnight and hybridize a minimum of 24 hours, up to 48 or more. Probes can be re-used multiple times and often you will get less background with re-used probes. For washes, doing more can't hurt and duration of these washes are just a suggestion, so it is not necessary to be that strict about it. To reduce the stringency of washes, use a higher salt concentration than 0.05x SSC (such as 0.2x SSC), which can speed up development time but may cause an increase in background. Developing is a key step, be patient and refresh substrate solution before it turns purple. Some probes come up in minutes (in which case you should try to develop it slower at 4 deg to achieve the best signal to noise) while others may take up to a week.
