# Supplementary figures and images for: Mesodermal gene expression during the embryonic and larval development of the articulate brachiopod Terebratalia transversa
Source: EvoDevo. 2015 Apr 11;6:10. doi: 10.1186/s13227-015-0004-8 (PMC4404124; doi:10.1186/s13227-015-0004-8)

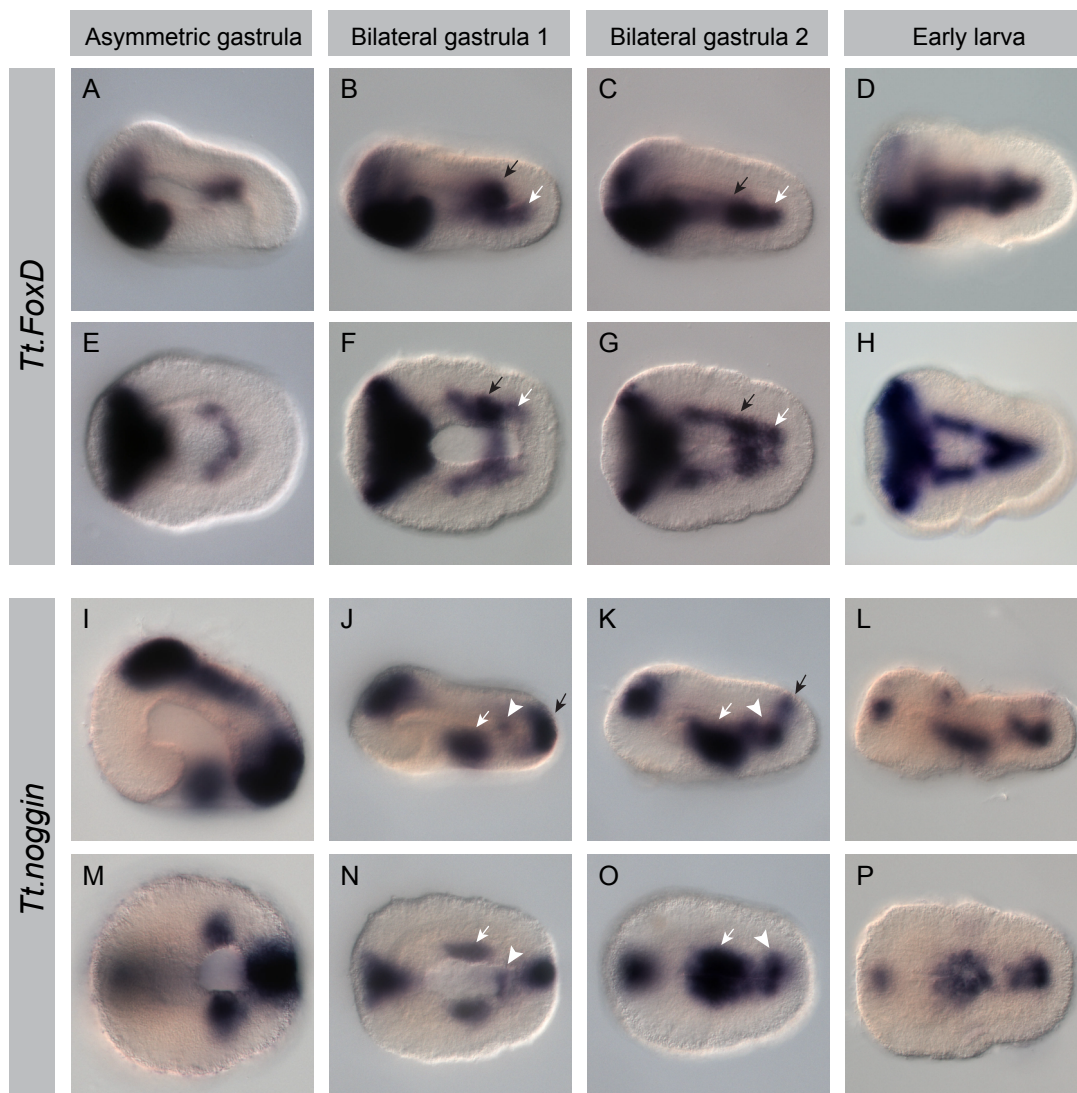

Supplement: Additional file 4: — Bayesian phylogenetic analysis of bHLH transcription factors. Bayesian phylogenetic analysis supports orthology assignments for Tt.MyoD, Tt.paraxis, and Tt.twist. [file 13227_2015_4_MOESM4_ESM.pdf]

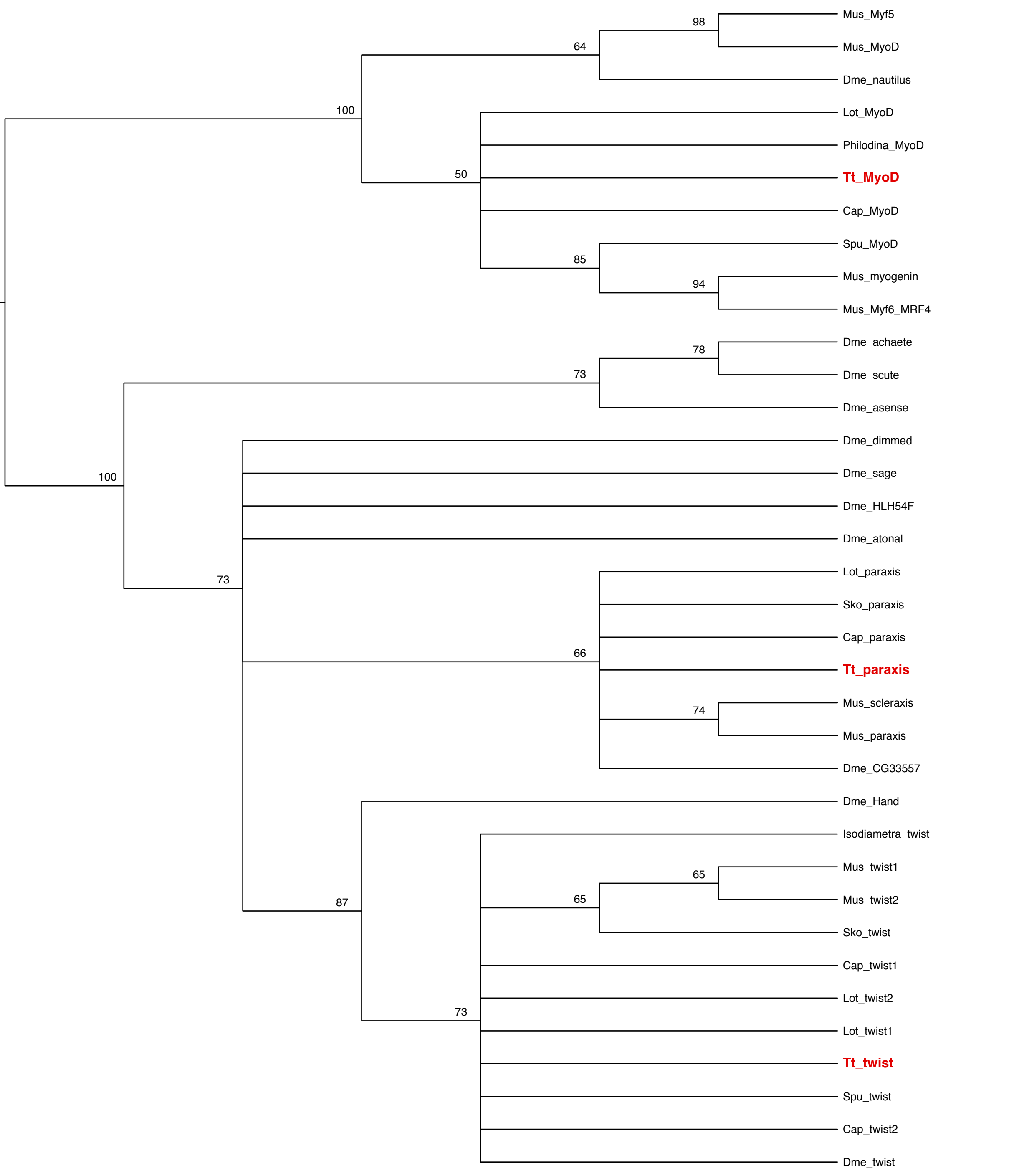

Supplement: Additional file 7: — Bayesian phylogenetic analysis of MADS-box transcription factors. Bayesian phylogenetic analysis supports orthology assignment for Tt.MEF2. [file 13227_2015_4_MOESM7_ESM.pdf]

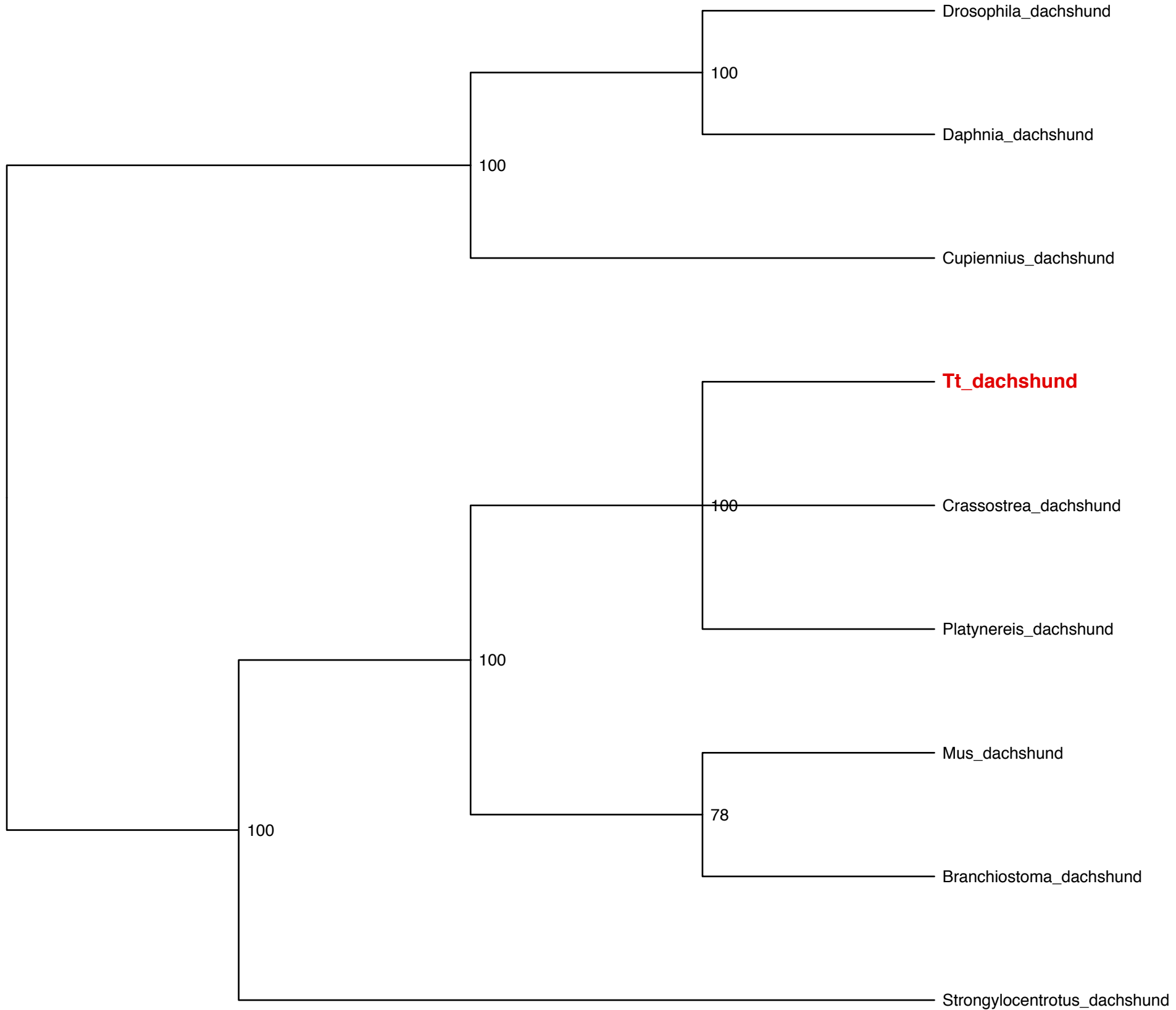

Supplement: Additional file 8: — Bayesian phylogenetic analysis of ANTP-class homeobox transcription factors. Bayesian phylogenetic analysis supports orthology assignment for Tt.Mox. [file 13227_2015_4_MOESM8_ESM.pdf]

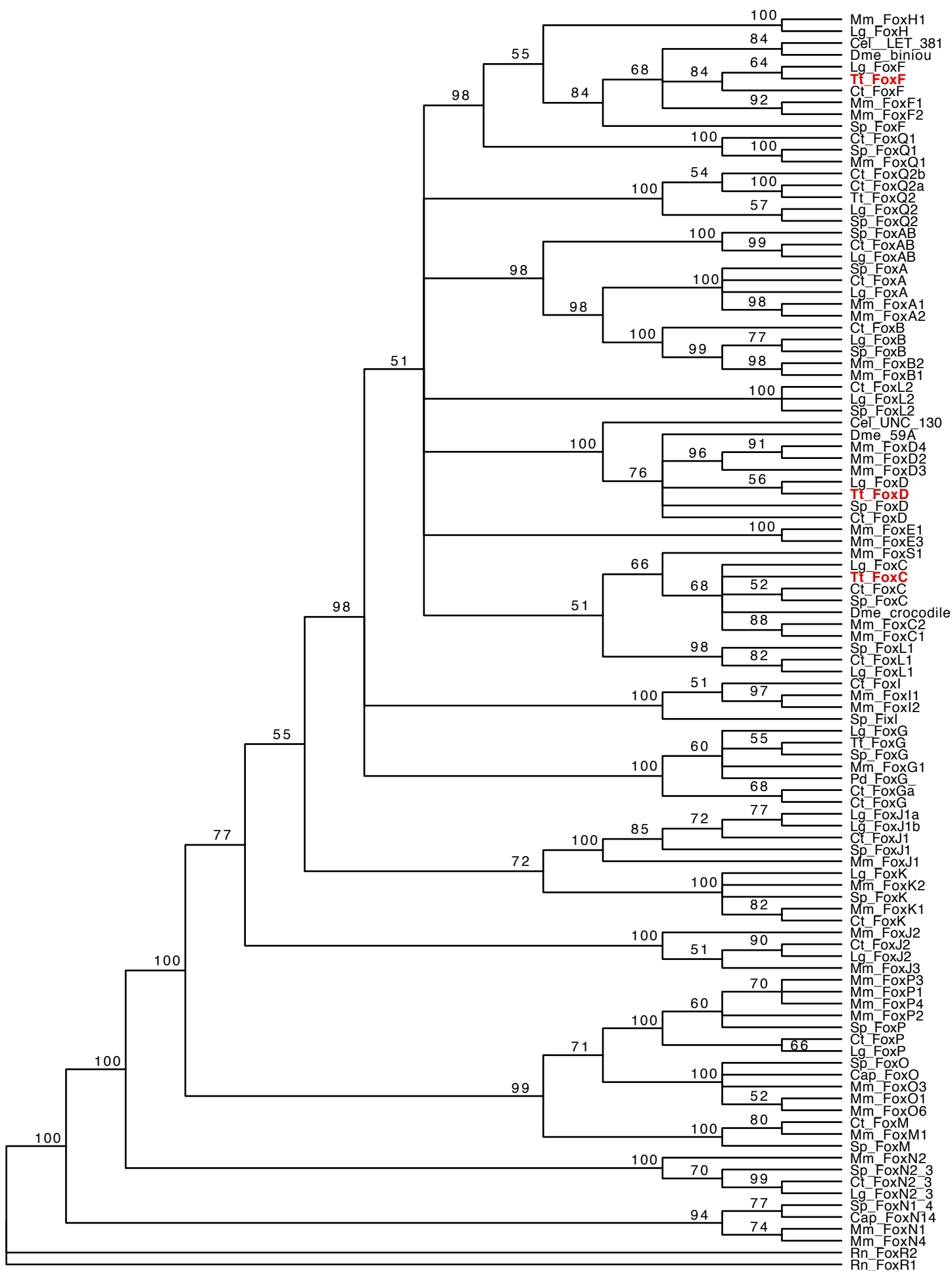

Supplement: Additional file 9: — Bayesian phylogenetic analysis of NK-class homeobox transcription factors. Bayesian phylogenetic analysis supports orthology assignment for Tt.NK1. [file 13227_2015_4_MOESM9_ESM.pdf]

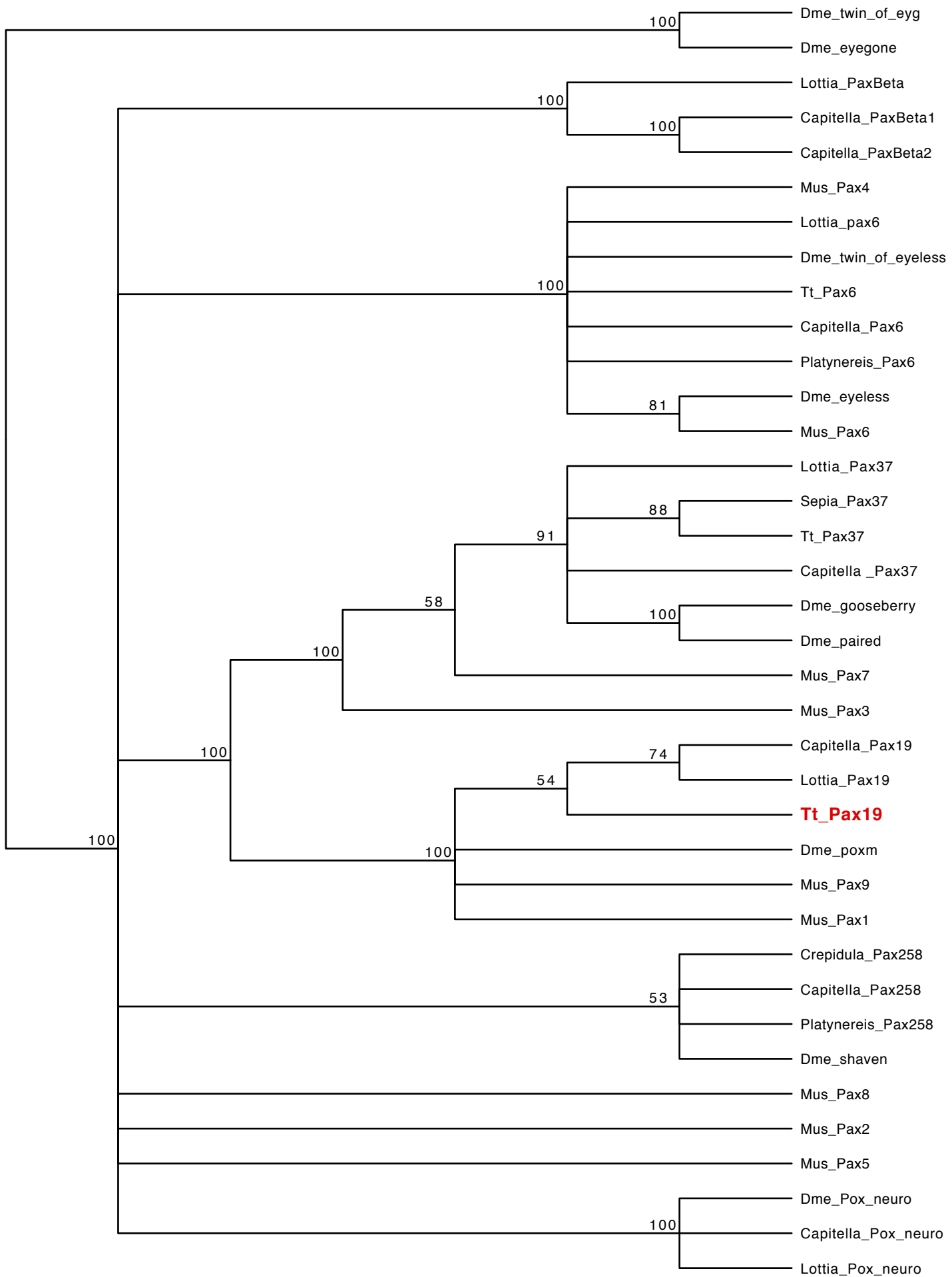

Supplement: Additional file 10: — Bayesian phylogenetic analysis of Paired box (Pax) transcription factors. Bayesian phylogenetic analysis supports orthology assignment for Tt.Pax1/9. [file 13227_2015_4_MOESM10_ESM.pdf]

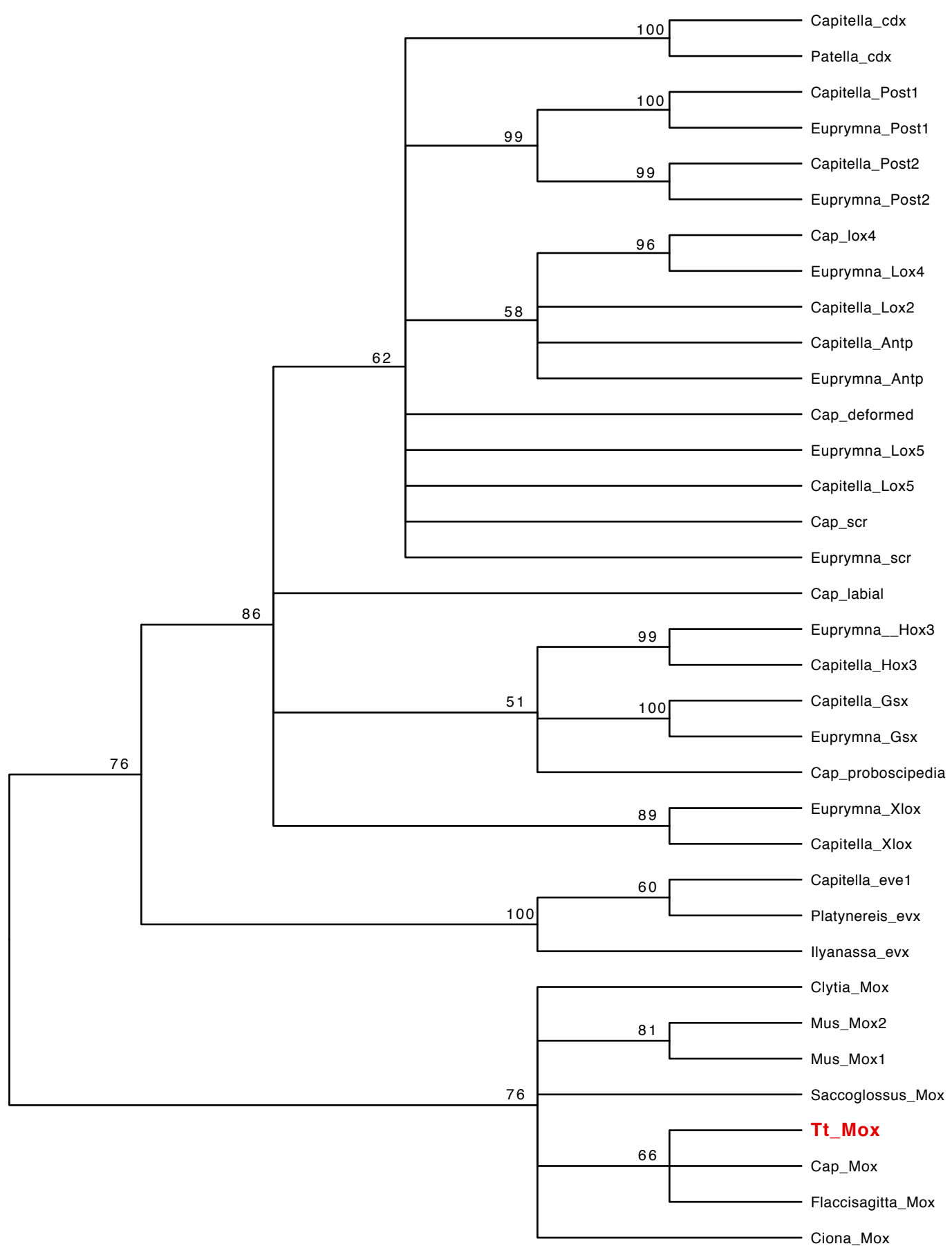

Supplement: Additional file 11: — Bayesian phylogenetic analysis of sine oculis (Six) class homeobox transcription factors. Bayesian phylogenetic analysis supports orthology assignment for Tt.Six1/2. [file 13227_2015_4_MOESM11_ESM.pdf]

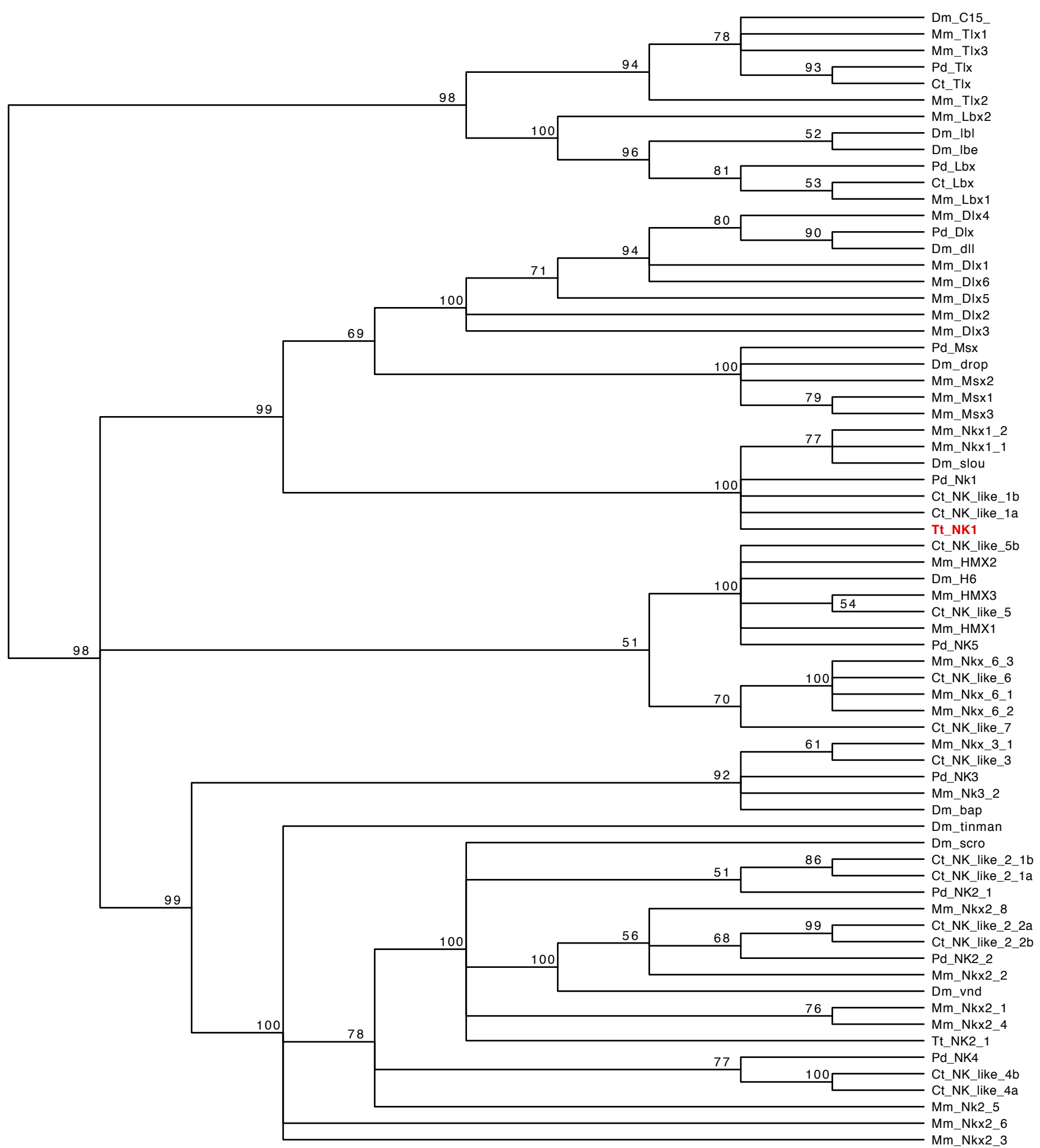

Supplement: Additional file 12: — Expression patterns of Tt.FoxD and Tt.noggin during the transition for gastrula to larval stages. All images are oriented with anterior to the left. Panels A-D and I-L are lateral views. Panels E-F and M-N are blastoporal views. Panels G-H and O-P are ventral views. For detailed descriptions of expression patterns, see text. (A-H) Tt.FoxD is expressed in a narrow band of cells at the border of the archenteron wall and roof in the asymmetric gastrula and transitions ventrally in the bilateral gastrula (black arrows). A second region of mesodermal expression develops in two ventrolateral posterior bands, which converge medially as the blastopore closes (white arrows). (I-P) Domains of Tt.noggin in the lateral regions of the blastopore lip of the asymmetric gastrula invaginate to contribute to the ventromedial mesoderm as the blastopore closes in the bilateral gastrula (white arrows). Expression in the posterior of the blastopore lip shifts to the dorsal ectoderm (black arrows), while a second domain of posterior mesodermal expression forms medially in the region that will form the pedicle lobe (white arrowheads). [file 13227_2015_4_MOESM12_ESM.pdf]
